# Biodegradable manganese engineered nanocapsules for tumor-sensitive near-infrared persistent luminescence/magnetic resonance imaging and simultaneous chemotherapy

**DOI:** 10.7150/thno.59840

**Published:** 2021-07-25

**Authors:** Rui Zou, Junwei Li, Ting Yang, Yong Zhang, Ju Jiao, Ka-Leung Wong, Jing Wang

**Affiliations:** 1 Department of Nuclear Medicine, The Third Affiliated Hospital of Sun Yat-sen University, 600 Tianhe Road, Guangzhou, Guangdong 510630, P.R. China; 2 Ministry of Education Key Laboratory of Bioinorganic and Synthetic Chemistry, State Key Laboratory of Optoelectronic Materials and Technologies, KLGHEI of Environment and Energy Chemistry, School of Chemistry, Sun Yat-sen University, Sun Yat-Sen University, Guangzhou 510275, P.R. China; 3 Department of Chemistry, Hong Kong Baptist University, Hong Kong S.A.R. 999077, P.R. China

**Keywords:** biodegradability, hollow structure, persistent luminescence, multimodal imaging, chemotherapy

## Abstract

**Rationale:** Near-Infrared persistent luminescence (NIR-PL) nanomaterials that can continually emit low-energy photons after ceasing excitation has emerged as a new generation of theranostic nanoparticle drug delivery systems (NDDSs) for imaging-guided cancer therapy, which stems from their special ability to completely avoid tissue autofluorescence interference. However, unresponsive diagnostic capability, inefficient drug delivery, and poor biodegradability limit the efficacy of most reported NIR-PL-based NDDSs.

**Methods:** Herein, a multifaceted tumor microenvironment (TME)-degradable theranostic drug delivery nanocapsule based on an ultrasmall persistent phosphor with a hollow mesoporous manganese-doped, DOX-loaded silica shell (Mn-ZGOCS-PEG) is developed to overcome the above drawbacks.

**Results:** We demonstrate that the well-designed nanocapsule enables tumor-responsive controlled drug release with ameliorated therapeutic efficacy, TME-responsive autofluorescence interference-free NIR-PL tracing, and manganese-enhanced magnetic resonance (Mn-MR) monitoring for practical dual-modality image-guided antitumor treatment *in vivo*.

**Conclusion:** Our results indicate that Mn-ZGOCS-PEG nanocapsules enable tumor-targeting augmented chemotherapy under the guidance of TME-responsive dual-MR/NIR-PL-modality imaging *in vivo*. We believe that our work provides a new paradigm for the development of smart NIR-PL-based NDDSs with ultrasensitive multimodal diagnostic capability, enhanced anticancer effect, and efficient biodegradability.

## Introduction

Near-Infrared persistent luminescence (NIR-PL) nanomaterials have emerged as a promising and practical technology platform for time-resolved biosensing/bioimaging with high signal-to-noise ratios, given that their superlong NIR afterglows can disregard autofluorescence, diminish light scattering, and display deep tissue penetration of light 1-8. To pursue reciprocal and synergistic improvements of the detection sensitivity, spatial resolution, intrinsic biocompatibility, and treatment accuracy of image-guided therapy with NIR-PL nanomaterials integrating them with other imaging modalities (e.g. magnetic resonance imaging MRI) or engineering them with other therapeutic functionalities (e.g. with photodynamic therapy and controlled drug delivery/release) have become strategically all-rounded approaches in hot pursuit over the recent decade 9-21. Among the literature examples, the development of theranostic NIR-PL nanoparticle drug delivery systems (NDDSs) to enhance the *in vivo* efficacy of drugs, while allowing real-time monitoring of the biodistribution and target accumulation, has become one of the active NIR-PL research directions 22-28. That said, most of the NIR-PL NDDSs reported have demonstrated unresponsive imaging capability and very unsatisfactory drug delivery/controlled release efficiencies, plausibly resulting in poor therapeutic outcomes; their inferior biodegradabilities and so their accumulations within the body, have also raised significant challenges and concerns for further clinical translations. Hence, there is an urgent demand for contriving a smart NIR-PL theranostic NDDS that simultaneously enables highly efficient drug delivery/controlled release *in vivo* and excellent *in vivo* biodegradation/clearance, while affording “on-demand” *in vivo* multimodal imaging.

Inspired by the facts that different from normal tissues, a characteristically acidic and reducing tumor microenvironment (TME) can be found around solid tumors 29-33, and that the -Mn-O- bonds are sensitive to the TME 34-38, we herein develop an intelligent biodegradable theranostic NDDSs *in vivo* based on ultrasmall NIR-PL nanoparticles (NPs) with hollow mesoporous Mn-doped silica shells (Mn-ZGOCS-PEG) for imaging-guided tumor therapy. By our rational design, dissociation of -Mn-O- bonds under TME can simultaneously promote the biodegradation of Mn-ZGOCS-PEG, thereby triggering a burst release of the drug; the subsequent Mn extraction can then significantly improve the T_1_-weighted magnetic resonance (MR) imaging. Besides, the previously quenched persistent luminescence of NIR-PLNPs can be recovered for TME-sensitive autofluorescence-free diagnosis due to the disorganization of Mn-ZGOCS-PEG nanocapsules. Our* in vivo* comprehensive experiments substantiate that the Mn-ZGOCS-PEG nanocapsules not only exhibit high tumor-inhibition efficacy without observably adverse effects but also good biodegradability for safe elimination away from the body. As such, our intelligent biodegradable NIR-PL theranostic NDDSs can provide tumor-targeting augmented chemotherapy under the guidance of TME-responsive dual-MR/NIR-PL-modality imaging *in vivo*, showcasing huge translational potential in precise cancer therapy.

## Materials and methods

### Materials

Zn(CH_3_COO)_2_·2H_2_O, Cr(CH_3_COO)_3_, SnCl_4_, ammonium hydroxide (NH_3_·H_2_O), Hexadecyl trimethyl ammonium bromide (CTAB), Tetraethyl orthosilicate (TEOS), disodium maleate, MnSO_4_·H_2_O, and glutathione (GSH) are purchased from Aladdin (Shanghai, China). Gallium (III) nitrate solution is purchased from Alfa Aesar (Shanghai, China). Methoxy PEG Silane (*M*_W_ = 2000) is ordered from SHANGHAI ZZBIO Co., Ltd (Shanghai, China). Doxorubicin hydrochloride is obtained from Dalian Meilun Biological Technology Co., Ltd (Dalian, China). All the initial chemicals are of analytical grade and used without further purification.

### Characterizations

The morphology and structure of the samples are characterized on a Field-emission scanning electron microscopy (SEM, Bruker Gemini500). Transmission electron microscopy (TEM) micrograph, high-resolution TEM (HRTEM) micrograph, energy disperse spectroscopy (EDS) and high-angle annular dark-field scanning (HAADF-STEM) images are conducted on FEI Tecnai G2 F30 (300 kV). The phases of nanoparticles are determined by using powder x-ray diffraction (XRD, D8 ADVANCE) under Cu Kα_1_ radiation (λ = 1.5405 Å). Surface area and pore-size distribution analyses are performed on a gas adsorption analyzer (ASAP 2020M). X-ray photoelectron spectroscopy (XPS) spectrum is acquired on a Thermo Fisher Scientific X-ray photoelectron spectroscopy (K-Alpha^+^) under Al Kα radiation. Photoluminescence excitation, emission spectra, and persistent luminescence signals are collected on an FLS1000 Fluorescence Spectrometer (Edinburgh Instruments). UV-vis absorption spectra are recorded on a Cary 5000 UV-vis-NIR spectrophotometer (Varian). The hydrodynamic diameter and zeta potential analysis are performed on a nanoparticle size-zeta potential and molecular weight analyzer (Brookhaven).

### Synthesis of ZnGa_2_O_4_:Cr^3+^, Sn^4+^@SiO_2_@mSiO_2_ (ZGOCS@SiO_2_@mSiO_2_) nanoparticles

ZnGa_2_O_4_:Cr^3+^, Sn^4+^@SiO_2_ (ZGOCS@SiO_2_) nanoparticles are synthesized according to our previous method 11. Then 0.1 g of ZGOCS@SiO_2_ nanoparticles are added into a mixture of 56 mL of deionized water, 18 mL of ethanol, and 1.1448 g of CTAB. After sonication for 2 h, 0.1 mL of ammonium hydroxide is added into the above solution. Thirty minutes later, 0.2 mL of TEOS is slowly added into the mixture and stirred for 4 h at room temperature. The resultant nanoparticles are collected by centrifugation and washed with deionized water and ethanol three times. To remove the CTAB template, the obtained nanoparticles are annealed at 550 °C for 5 h with a slow heating rate of 2 °C/min. Finally, the ZGOCS@SiO_2_@mSiO_2_ nanoparticles are obtained after cooling down to room temperature.

### Synthesis of manganese-engineered ZnGa_2_O_4_:Cr^3+^, Sn^4+^ near-infrared persistent luminescence hollow nanoparticles (Mn-ZGOCS)

Briefly, 50 mg of ZGOCS@SiO_2_@mSiO_2_ nanoparticles, 160 mg of MnSO_4_·H_2_O, and 200 mg of disodium maleate are added to a beaker having 20 mL of deionized water. After sonication for 2 h, the mixture is transferred into a 40 mL Teflon-lined stainless-steel autoclave. Then, the autoclave is sealed and maintained in a hydrothermal condition at 180 °C for 12 h. The resulting Mn-ZGOCS nanoparticles are washed three times with deionized water and ethanol, separated by centrifugation, and finally dried at 60 °C for 12 h.

### Synthesis of PEGylated Mn-ZGOCS (Mn-ZGOCS-PEG) nanocapsules

PEG functionalization of Mn-ZGOCS nanocapsules is conducted via a typical silane coupling process. Firstly, 100 mg of methoxy PEG silane (*M*_W_ = 2000) is initially dispersed into 100 mL of ethanol solution by sonication, and then 20 mg of Mn-ZGOCS nanocapsules are added. To ensure the reaction completeness, the mixture is gently stirred at 60 °C for 24 h. After that, PEGylated Mn-ZGOCS are collected by centrifugation and washed three times with deionized water and ethanol to remove the residual PEG. PEGylated ZGOCS@SiO_2_@mSiO_2_ (ZGOCS@SiO_2_@mSiO_2_-PEG) nanoparticles are prepared by a similar procedure.

### In Vitro Degradation Experiment

Typically, 5 mg of Mn-ZGOCS nanocapsules are added into 50 mL of PBS solution (pH 7.4) without or with GSH (5.0 mM and 10.0 mM) and set in a water bath at 37 °C with magnetic stirring (250 rpm). At timed intervals, a small amount of degradation solution is taken out for TEM observations and the ICP-OES test. Similarly, PBS solutions with different pH values (pH 7.4 and 5.0) are adopted to investigate the pH influence on degradation.

### Animal Tumor Models

5-6 weeks old nude mice (BALB/c, male, and weight ∼20 g) are purchased from Beijing Vital River Laboratory Animal Technology Co., Ltd. Then, LNCaP cells (1 × 10^6^ cells) are subcutaneously injected into mice in the right lower limb region to establish a tumor model. All experiments involving mice are performed under the guidelines of the Institutional Animal Care and Use Committee of the South China Agricultural University.

### In vitro and in vivo T_1_-weighted MR imaging

*In vitro* T_1_-weighted MR images and relaxation time measurements are performed on a 3.0 Tesla MRI instrument (MAGNETOM Verio, Siemens Medical Solution, Erlangen, Germany) with a head coil. Mn-ZGOCS-PEG nanocapsules (manganese concentrations: 0, 0.125, 0.25, 0.5, 1 and 2 mM) are dispersed in two different PBS solutions (pH 7.4, pH 5.0 with 10 mM GSH). T_1_ relaxation data are obtained by using a T_1_ map sequence as we previously described 39. Through the curve fitting of relaxation time 1/T_1_ (s^-1^) vs the molar concentration of manganese (mM), the r_1_ relaxivity values are acquired from the slope of the fitting line.

For *in vivo* T_1_-weighted MRI, the LNCaP tumor-bearing mice are anesthetized, and then intravenously administrated with Mn-ZGOCS-PEG nanocapsules (0.5 mg·mL^-1^, 200 µL). MR Images are acquired by a TSE sequence as we previously reported 39.

### In vitro and in vivo NIR persistent luminescence imaging

*In Vitro* and *in vivo* NIR persistent luminescence imaging is performed using an IVIS Lumina LT Series III imaging system (Caliper Lifesciences, USA). For *in vitro* NIR persistent luminescence imaging, Mn-ZGOCS-PEG nanocapsules are dispersed in two different PBS solutions (pH 7.4, pH 5.0 with 10 mM GSH). At timed intervals, NIR persistent luminescence images are acquired after 30 s irradiation of 254 nm UV light (6 W) with an exposure time of 5 s.

For *in vivo* NIR persistent luminescence imaging after intratumor injection, 20 µL of Mn-ZGOCS-PEG nanocapsules (10 mg/mL) are intratumorally administrated into the LNCaP tumor-bearing mouse in situ. Then NIR persistent luminescence images are acquired after intratumoral administration for 5, 30, 60, 120, 180, and 240 min. Before acquiring luminescence images, in situ excitation with a white LED light (1800 lm, NITECORE EC30) is performed on the mouse for 2 min. The exposure time is set as 30 s.

For *in vivo* NIR persistent luminescence imaging after intravenous injection, 200 µL of Mn-ZGOCS-PEG nanocapsules (2 mg/mL) are intravenously administrated into the LNCaP tumor-bearing mouse in situ. Then NIR persistent luminescence images are acquired after intravenous injection for 15, 60, 120, and 180 min. Before acquiring luminescence images, in situ excitation with a white LED light (1800 lm, NITECORE EC30) is performed on the mouse for 1 min. The exposure time is set as 120 s.

### Ex vivo biodistribution analysis

The mice are sacrificed at the time point of 6 h and 24 h after intravenous injection with 200 µL of Mn-ZGOCS-PEG nanocapsules (2 mg/mL). The main organs, including heart, liver, spleen, lung, kidney, small intestine, large intestine, stomach, and tumor tissues, are taken out and melt with concentrated nitric acid. The concentrations of element Zn and Mn within the organs and tumor are analyzed by ICP-OES test, respectively.

### Metabolism Research

The urine and faeces of mice are collected at the time point of 6 h, 24 h, and 48 h after intravenous injection of 200 µL of Mn-ZGOCS-PEG nanocapsules (2 mg/mL), respectively. The concentrations of element Zn and Mn within the urine and faeces are analyzed by ICP-OES test, respectively.

### Cellular GSH Assay

LNCaP cells (1 × 10^4^ cells) are seeded into 6-well plates and cultured for 24 h at 37 °C, 5% CO_2_. Then, the cells are co-incubated with various concentrations of Mn-ZGOCS-PEG (0, 25, 50, 100 µg·mL^-1^) in the culture medium for 12 h. Before use, the cells are collected by centrifugation and washed with PBS several times. A GSH assay kit (Beyotime, China) is used to evaluate the GSH content. After 30 min of coincubation, the absorbance values of each well at 412 nm are monitored to determine the GSH content by a microplate reader (Model 680, BIO-RAD).

### Loading of DOX

5 mg of Mn-ZGOCS-PEG nanocapsules are dispersed into 20 mL of DOX PBS solution (pH 7.4, 0.5 mg·mL^-1^). After 24 h of stirring, the DOX loaded Mn-ZGOCS-PEG (DOX-Mn-ZGOCS-PEG) nanocapsules are centrifugated and wished with PBS. when the supernatant becomes colorless, the absorbances of the supernatant and washed solutions are tested by UV-vis measurement. The DOX loading efficiency is quantified as follows: (weight of loaded DOX in Mn-ZGOCS-PEG/weight of DOX loaded Mn-ZGOCS-PEG) × 100%. A similar procedure is used to prepare DOX loaded ZGOCS@SiO_2_@mSiO_2_-PEG (DOX-ZGOCS@SiO_2_@mSiO_2_-PEG) nanoparticles and calculate the corresponding DOX loading efficiency.

### In vitro DOX release

To assess *in vitro* release of DOX, as prepared DOX-Mn-ZGOCS-PEG nanocapsules are added into 5 mL of PBS solutions at different GSH concentrations (0, 5.0, and 10.0 mM) or PBS solutions with different pH values (7.4 and 5.0). Then, the testing solutions are put into a water bath at 37 °C with magnetic stirring. At timed intervals, the supernatant is collected by centrifugation and 5 mL of fresh PBS is added to the testing solution. The amount of released DOX is estimated by monitoring the absorbance of taken supernatants at 480 nm.

### In vitro cellular uptake

LNCaP cells (1 × 10^4^ cells) are seeded into a culture plate and cultured overnight at 37 °C in an atmosphere with 5% CO_2_. Then, the cells are incubated with DOX-Mn-ZGOCS-PEG (50 µg·mL^-1^) for different incubation times (0.5, 1, and 3 h). After washing with PBS (pH = 7.4) several times, the cells are stained with 4', 6-diamidino-2-phenylindole (DAPI) for 10 min. At last, a confocal laser scanning microscopy (CLSM) instrument (LSM710, Carl Zeiss) is used to analyze the cellular uptake of samples.

### In vitro cell experiments

CCK-8 assay is applied to assess the *in vitro* cytotoxicity of the samples. For cell toxicity test, LNCaP cells (1 × 10^4^ cells) are seeded into a 96-well plate and cultured in an atmosphere with 5% CO_2_ for 24 h (37 °C). After that, the cells are co-incubated with various concentrations of Mn-ZGOCS-PEG (0, 25, 50, 100 µg·mL^-1^) in the culture medium for 24 h. Then the culture medium is replaced by a mixture solution containing fresh media (90 µL) and CCK-8 solution (10 µL) and incubated for another 2 h. Finally, the absorbance values of each well at 450 nm are monitored by a microplate reader (Model 680, BIO-RAD). The cytotoxicity of the sample is determined by the viability percentage of cells in the treated group to the control group.

For *in vitro* chemotherapy, LNCaP cells (1 × 10^4^ cells) are seeded into a 96-well plate and cultured at 37 °C in a humid incubator (5% CO_2_) for 24 h. Free DOX, DOX- ZGOCS@SiO_2_@mSiO_2_-PEG and DOX-Mn-ZGOCS-PEG are dispersed into the culture medium at the equivalent DOX contents, and the DOX concentrations are 0, 0.31, 0.63, 1.25, 2.5, 5, 10, 20 and 40 µg·mL^-1^. After 24 h of co-incubation, the culture medium is replaced by a mixture solution containing fresh media (90 µL) and CCK-8 solution (10 µL) and cultured for another 2 h. At last, a microplate reader is used to record the absorbance value at 450 nm. The cytotoxicity of samples is determined by the viability percentage of cells in the treated group to the control group.

### In vitro observation of dead cells

LNCaP cells (1 × 10^4^ cells) are seeded into a culture plate and cultured in an atmosphere with 5% CO_2_ for 24 h (37 °C). After that, the cells are incubated with PBS, DOX, DOX-ZGOCS@SiO_2_@mSiO_2_-PEG, and DOX-Mn-ZGOCS-PEG with the equivalent contents of DOX for 4 h. After three times of washing with PBS (pH = 7.4), the cells are stained by propidium iodide (PI) for 10 min. Finally, the fluorescence images of cells are recorded by a CLSM instrument (LSM710, Carl Zeiss).

### Blood Hemolysis

3 ml of mice whole blood is centrifuged (2500 rpm/min, 5 min) and washed with PBS for several times to collect red blood cells (RBCs). The collected RBCs are resuspended in 24 mL of PBS before use. Then, 0.2 mL of RBCs PBS was mixed with 0.8 mL of working solutions (DOX, DOX-ZGOCS@SiO_2_@mSiO_2_-PEG nanoparticles, and DOX-Mn-ZGOCS-PEG nanocapsules PBS solutions) at the equivalent DOX contents, and the DOX concentrations are 3.90625, 7.8125, 15.625, 31.25, 62.5, 125, and 250 µg·mL^-1^. PBS and deionized water are set as negative control and positive control, respectively. After incubation at 37 ℃ for 2 hours, 0.1 mL of the supernatants are transferred to a 96-well plate and the absorbances at 561 nm are monitored by a microplate reader. The hemolytic percentages are estimated by the following formula: Hemolysis (%) = (OD_samples_- OD_(-)control_) / (OD_(+)control_- OD_(-)control_).

### In Vivo Chemotherapy

When the tumor size is 6-8 mm (4 days after injection), the tumor-bearing mice are randomly divided into four groups (4 per group) and intravenously administrated with PBS (control), free DOX, DOX-ZGOCS@SiO_2_@mSiO_2_-PEG, and DOX-Mn-ZGOCS-PEG (200 μL, 0.25 mg·mL^-1^ as DOX), respectively. In the first 6 days, the mice are administrated with the mentioned nanoparticles every 2 days. Half a month after chemotherapeutic administration, the volume of the tumor is monitored every 2 days and estimated according to the following equation: the tumor volume = width^2^ × length /2.

### Histological Examination

After 14 days of the experiment, the heart, liver, spleen, lung, kidney, and tumor tissues from the representative mouse in each group are sectioned into slices for H&E and TUNEL staining analysis. The obtained stained slices are observed by an optical microscope.

## Results and Discussion

### Design, synthesis, and characterization of Mn-ZGOCS-PEG nanocapsules

**Scheme 1:**
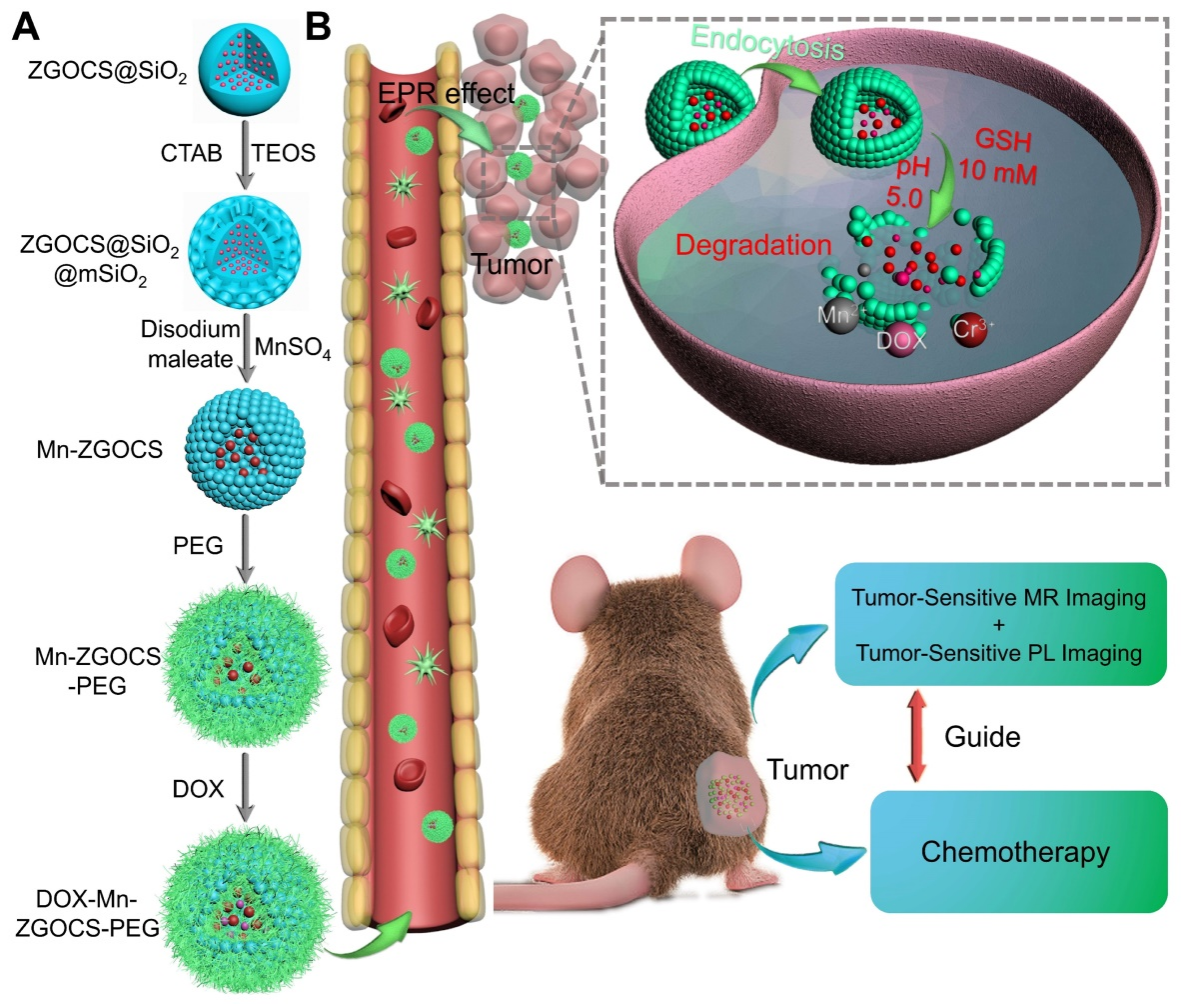
Schematic illustration of DOX-Mn-ZGOCS-PEG nanocapsules for tumor-sensitive dual-modality bioimaging and chemotherapies. (A) Design and step-by-step fabrication of DOX-Mn-ZGOCS-PEG nanocapsules and the subsequent anticancer drug loading. (B) Transport within a blood vessel, subsequent EPR effect mediated tumor accumulation, cellular uptake, biodegradation, and tumor microenvironment enhanced theranostic functions of DOX-Mn-ZGOCS-PEG nanocapsules.

The procedure for the synthesis of hollow DOX-loaded Mn-ZGOCS-PEG nanocapsules is illustrated in Scheme 1A. Firstly, highly dispersed and uniform ZGOCS@SiO_2_ nanoparticles are prepared according to our previous procedure as the NIR-PL source because of their excellent PL properties and white light rechargeable ability 11. Given that the encapsulation of ZGOCS nanoparticle almost occupies all the cavities in the mesoporous silica, a uniform mesoporous silica (mSiO_2_) shell is subsequently coated onto the surface of ZGOCS@SiO_2_ nanoparticles to facilitate the interaction between manganese and silica 22, 40-42. After that, a surface-catalyzed dual template method is employed to synthesize manganese-engineered hollow ZGOCS NIR-PL nanocapsules (Mn-ZGOCS) by an *in-situ* transformation of the mesoporous silica into the manganese-doped silica shell. To improve their water solubility and physiological stability, as-prepared Mn-ZGOCS nanocapsules are further surface-modified with silane-polyethylene glycol (PEG). Finally, anticancer drug doxorubicin (DOX) is loaded into the hollow structure of Mn-ZGOCS-PEG nanocapsules, forming DOX-Mn-ZGOCS-PEG, which will be used for chemotherapeutic purpose upon releasing. Here, an elaborate engineered tumor-sensitive dual-modal theranostic nano-capsules can be obtained. Scheme 1B depicts the blood circulation of DOX-Mn-ZGOCS-PEG nanocapsules, the subsequent intracellular delivery, the biodegradation, and the enhanced theranostic functions. DOX-Mn-ZGOCS-PEG nano-capsules can accumulate at tumor tissues via the typical enhanced permeability and retention (EPR) effect through the blood circulation. Once DOX-Mn-ZGOCS-PEG nanocapsules are endocytosed into tumor tissues, either the mild acidic or the reductive microenvironment of tumor issue would trigger the release of Mn ions and the simultaneous biodegradation of DOX-Mn-ZGOCS-PEG nanocapsules. Tactfully, such biodegradation of DOX-Mn-ZGOCS-PEG nanocapsules and the release of Mn ions would facilitate the release of the loaded DOX, thereby enhancing the contrast effect of T_1_-weighted MR imaging. Moreover, the disorganization of manganese-doped silica shell can also reduce the luminescence quenching of the ZGOCS NIR-PL nanoprobes due to the Förster resonance energy transfer (FRET) effect, resulting from the overlap of the emission of the ZGOCS nanoparticles and absorption of manganese species, and then in the activation of autofluorescence-free PL bioimaging. Through these elaborate designs, biodegradation and tumor-specific enhanced theranostic functions can be concurrently assembled into an all-in-one DOX-Mn-ZGOCS-PEG nanocapsule, which lends itself to a highly promising nanosystem for accurate diagnosis and treatment of cancers.

**Figure 1:**
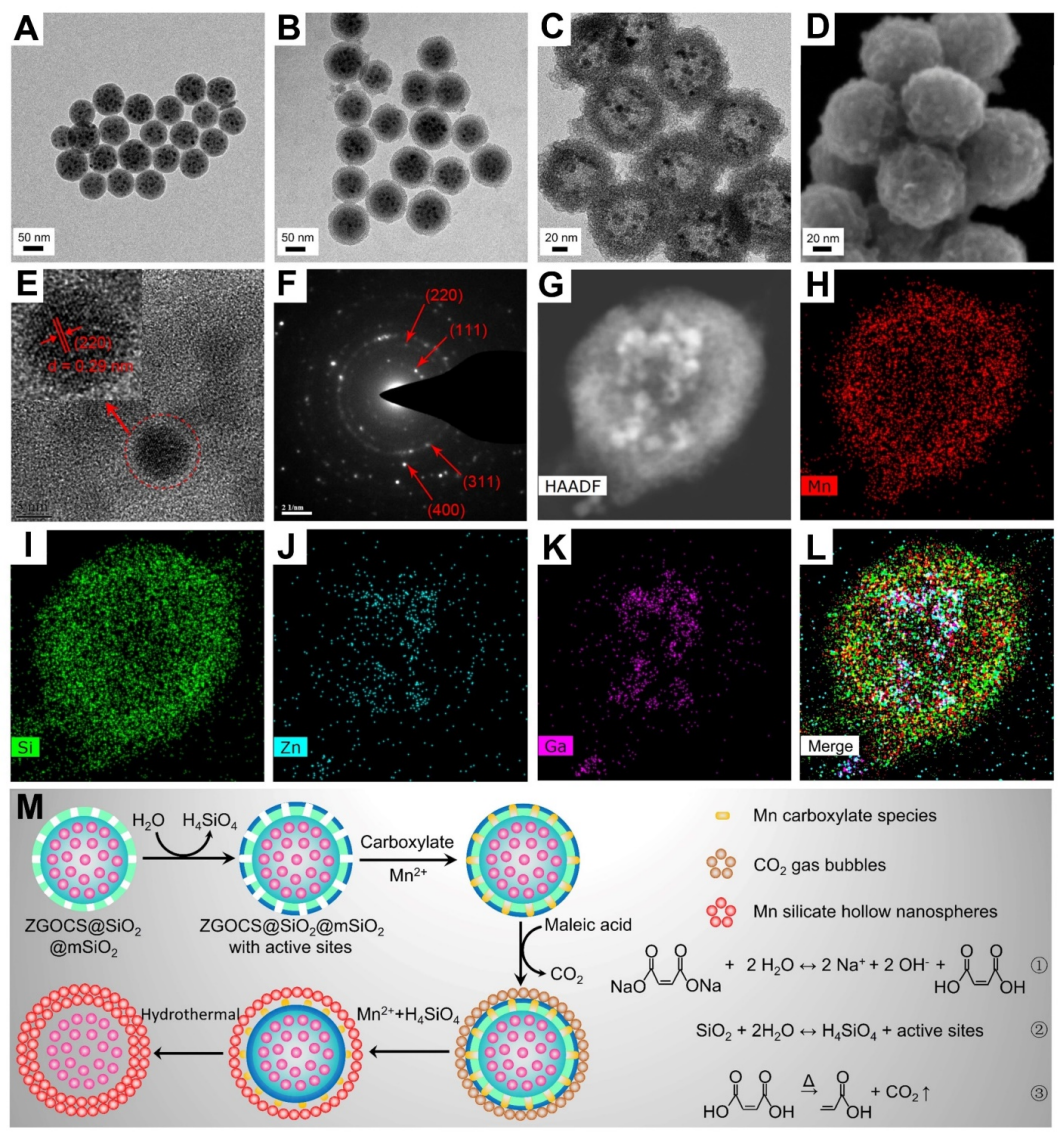
(A)-(C) TEM images of ZGOCS@SiO_2_ core nanoparticles, core-shell ZGOCS@SiO_2_@mSiO_2_ nanoparticles, and Mn-ZGOCS nanocapsules. (D) HRSEM image (E) HRTEM image (F) SAED pattern of Mn-ZGOCS nanocapsules. (G)-(I) HAADF-STEM image and corresponding elemental mapping of Mn-ZGOCS nanocapsules (H) Mn, (I) Si, (J) Zn, (K) Ga, (L) merged. (M) Schematic illustration of the formation process of Mn-ZGOCS nanocapsules: (Step A) the reaction between disodium maleate and water result in a weakly alkalescent solution environment (reaction ①), (Step B) silica dissolution to release H_4_SiO_4_ (reaction ②), (Step C) CO_2_ gas bubbles resulting from carboxylate decomposition serve as a soft template for the deposition of Mn silicate (reaction ③), (Step D) exhaustion of silica to form sphere-stacked hollow nanocapsules with ZGOCS nanoparticles interior.

The transmission electron microscope (TEM) image in Figure 1A shows that the as-prepared ZGOCS@SiO_2_ nanoparticles are uniform, and monodisperse, being spherical with a particle diameter of about 70 nm (Figure S1A). ZGOCS NIR-PLNPs (dark spots within the nanospheres) with a particle diameter of about 7 nm are distributed inside the nanopores of the mesoporous silica (Figure S2A). After coating with mesoporous silica, ZGOCS@SiO_2_@mSiO_2_ nanoparticles still retain the uniformity, the monodispersity, and the spherical morphology, except with a larger particle diameter of about 100 nm (Figure 1B and Figure S1B). The uniform mesoporous silica shell possesses wormhole-like channels and the thickness of such shell is about 15 nm (Figure S2A). The TEM image of hollow structure Mn-ZGOCS nanocapsules reveals the spherical morphology of the final obtained product, and the size of Mn-ZGOCS nanocapsules is about 100 nm. (Figure 1C and Figure S1C). The thickness of such manganese-doped silica shell is measured to be about 20 nm, which is composed of numerous smaller nanobubbles (Figure S2B), and the formation of these nanobubbles can be further confirmed by the high-resolution scanning electron microscopy (HRSEM) image (Figure 1D). HRTEM image (Figure 1E) taken at the interior of selected Mn-ZGOCS nanocapsules manifests that the interplanar spacing is 0.29 nm, which corresponds to the (220) plane of cubic phase ZnGa_2_O_4_, indicating ZGOCS NIR-PLNPs are involved in the interior of nanocapsules. The selected area electron diffraction (SAED) pattern also reveals the existence of a cubic phase ZnGa_2_O_4_ (Figure 1F). Through the high-angle annular dark-field scanning TEM (HAADF-STEM)-based elemental mapping (Figure 1G-L) and energy disperse spectroscopy (EDS) spectrum (Figure S3), a distinctive ZGOCS core@void@Mn-doped silica shell configuration can be further confirmed. To decipher the formation of these hollow nanocapsules, a surface-catalyzed dual templating process is summarized in Figure 1M. First, in the weakly alkaline hydrothermal environment provided by disodium maleate (Figure 1M, reaction 1), a small amount of the mSiO_2_ is hydrolyzed to form H_4_SiO_4_ (Figure 1M, reaction 2) 35, 43, 44. Simultaneously, active sites are generated on the surface of mSiO_2_ to adsorb manganese carboxylate species produced from disodium maleate decomposition (reaction 1). Under hydrothermal conditions, the carboxylate groups subsequently decompose into CO_2_ and other gaseous products (Figure 1M, reaction 3), which serve as soft templates for the deposition of manganese silicate generated from the ion-exchange of H_4_SiO_4_ species with Mn^2+^ ions 45, 46. Finally, with the consumption of SiO_2_, these reactions and the deposition process would stop, then form nanobubbles-stacked hollow nanocapsules, of which manganese silicate is deposited on the surface of nanobubbles and ZGOCS NIR-PLNPs are involved in the interior of nanocapsules.

**Figure 2:**
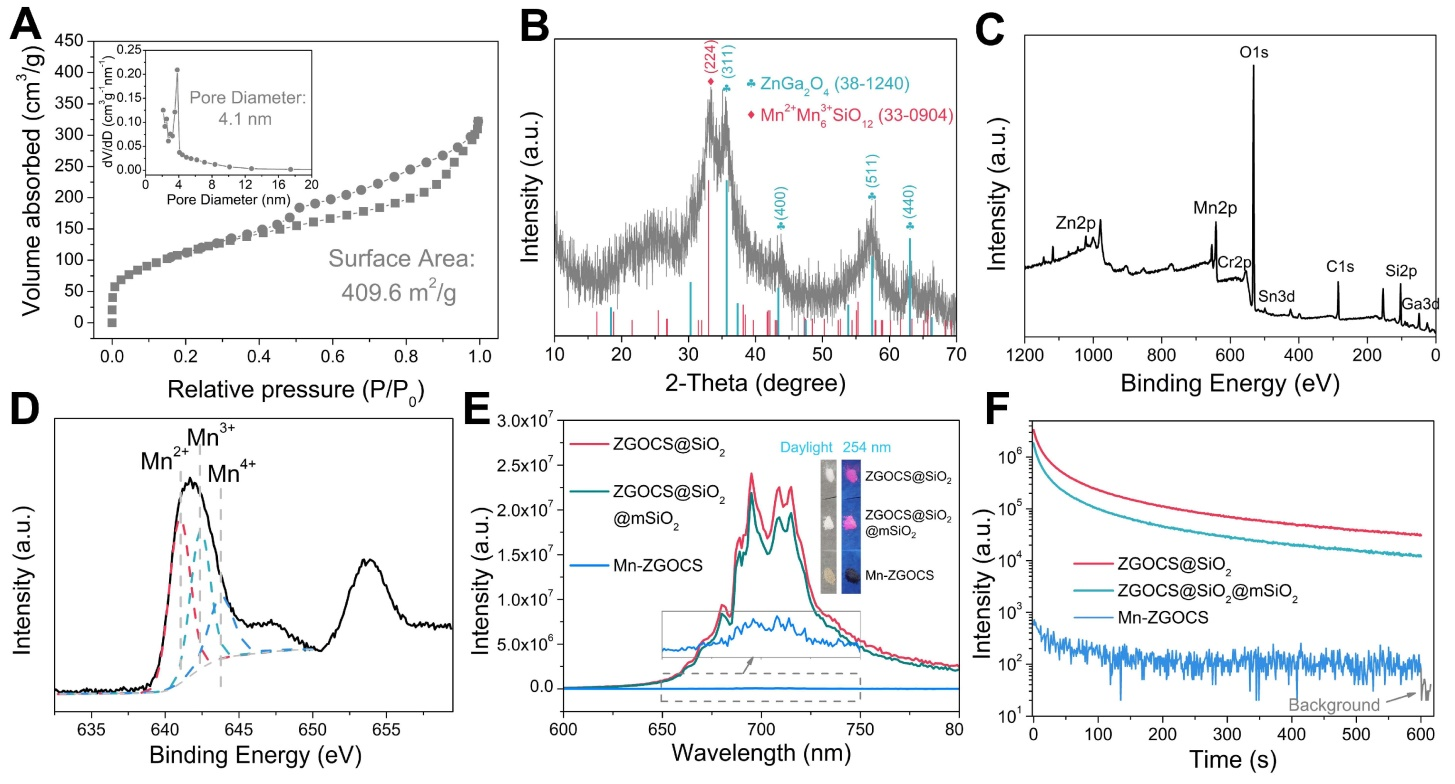
(A) N_2_ adsorption/desorption isotherm and pore size distributions (inset) of Mn-ZGOCS nanocapsules. (B) XRD pattern, (C) Full range XPS spectrum, and (D) High-resolution XPS spectrum of Mn-ZGOCS nanocapsules. (E) Emission (excitation at 254 nm) spectra of ZGOCS@SiO_2_ core nanoparticles, core-shell ZGOCS@SiO_2_@mSiO_2_ nanoparticles, and Mn-ZGOCS nanocapsules. The inset shows the corresponding digital photos of each sample under irradiation of 254 nm UV light. (F) NIR persistent luminescence decay curves of ZGOCS@SiO_2_ core nanoparticles, core-shell ZGOCS@SiO_2_@mSiO_2_ nanoparticles, and Mn-ZGOCS nanocapsules monitored at 695 nm after 5 min irradiation of 254 nm UV light.

To evaluate the mesoporous structure of Mn-ZGOCS nanocapsules, N_2_ adsorption-desorption analysis is conducted. As shown in Figure 2A. The Brunauer-Emmet-Teller (BET) surface area and Brunauer-Joyner-Halenda (BJH) average pore size of Mn-ZGOCS nanocapsules are determined to be 409.6 m^2^ g^-1^ and 4.1 nm (Figure 2A, inset), respectively, which is much higher than that of ZGOCS@SiO_2_@mSiO_2_ nanoparticles (250.9 m^2^ g^-1^ and 2.8 nm, Figure S4 and inset). The hollow structure of Mn-ZGOCS nanocapsules with mesoporous shells favors efficient drug loading and release. Figure 2B presented the X-ray diffraction (XRD) pattern of as-synthesized Mn-ZGOCS nanocapsules. Most of the diffraction peaks can be indexed to pure cubic-phase ZnGa_2_O_4_ (JCPDS NO. 38-1240) 47. Besides, a broadened peak at about 2*θ* = 33^o^ can be indexed to braunite-1Q (Mn^2+^Mn^3+^_6_SiO_12_, JCPDS NO. 33-0904), indicating the covalent bonding of the manganese species within the silica framework 35, 43. The X-ray photoelectron spectroscopy (XPS) in the full range proved the existence of Zn, Ga, O, Cr, Sn, Mn, C, and Si elements (Figure 2C), which is following the EDS testing result (Figure S2). The high-resolution Mn 2p^3/2^ XPS spectrum is further used to identify the valence state of Mn (Figure 2D). As shown, Mn 2p^3/2^ peak could be deconvolved into three peaks at 641.0, 642.4, and 643.8 eV, representing the existence of Mn^2+^, Mn^3+^, and Mn^4+^ species with the amount of 43.4%, 35.2%, and 21.4%, respectively 48. Next, the optical properties of ZGOCS@SiO_2_, ZGOCS@SiO_2_@mSiO_2,_ and Mn-ZGOCS nanocapsules are investigated. ZGOCS@SiO_2_ nanoparticles give NIR emission at 695 nm originated from the spin-forbidden ^2^E → ^4^A_2_ transition of Cr^3+^ ions in the octahedral site of a spinel structure (Figure 2E) and exhibit excellent NIR-PL (Figure 2F) 49, 50. Only a slight decrease stems from the optical properties of the ZGOCS@SiO_2_ nanoparticles after coating with mSiO_2_ (Figure 2E and F). Unlike ZGOCS@SiO_2_@mSiO_2_, both the emission intensity and persistent luminescence performance (intensity and duration) of the Mn-ZGOCS nanocapsules (Figure S5) are decreased due to the quenching effect resulting from the overlap of the luminescence emission of the ZGOCS nanoparticles and absorption of manganese species (Figure S6). The digital photos of ZGOCS@SiO_2_, ZGOCS@SiO_2_@mSiO_2,_ and Mn-ZGOCS powders under irradiation of 254 nm UV light provided a visual perception of this quenching effect (Figure 2E, inset). Furthermore, FT-IR spectra are collected to verify the modification of PEG on the Mn-ZGOCS nanocapsules. Compared with the Mn-ZGOCS nanocapsules, the FT-IR spectra of Mn-ZGOCS-PEG nanocapsules give two absorption bands at 1110 cm^-1^ (C-O stretching vibration of the PEG chains) and 2884 cm^-1^ (-CH_2_- stretching vibration of PEG chains), indicating the successful PEGylation of the Mn-ZGOCS nanocapsules (Figure S7). Besides, the initial hydrodynamic diameter and zeta potential of Mn-ZGOCS are 136.31 nm and -26.36 mV, respectively, and changed to be 181.76 nm and -13.25 mV, respectively, after PEGylation, further demonstrated that PEG is linked on the surface of Mn-ZGOCS nanocapsules (Figure S8).

### TME-responsive biodegradation of Mn-ZGOCS-PEG nanocapsules

**Figure 3:**
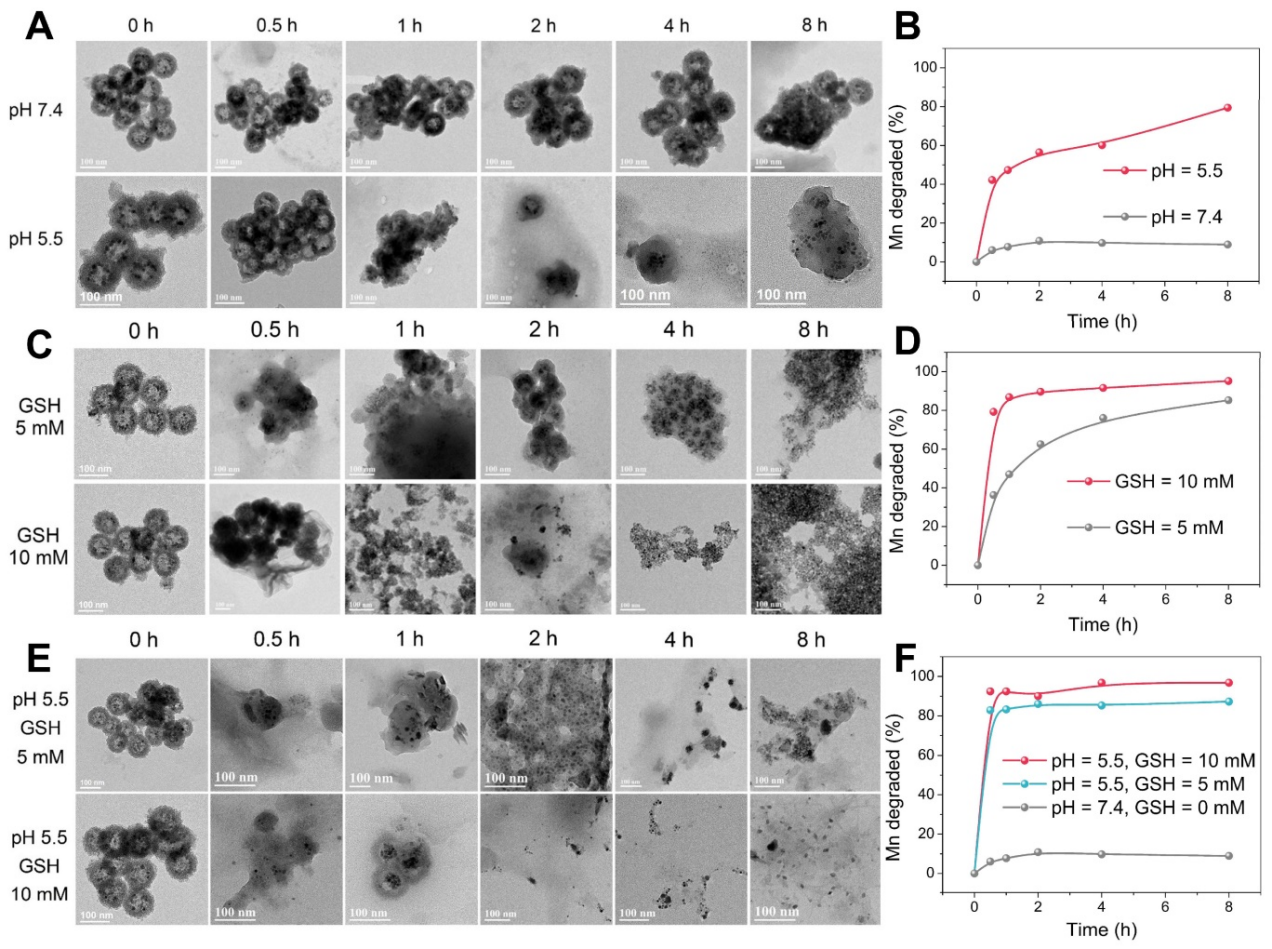
TEM images of Mn-ZGOCS nanocapsules and corresponding releasing profiles of biodegraded Mn ions after various periods of incubation in PBS (A, B) with different pH values, (C, D) with different GSH contents under neutral condition, and (E, F) with different GSH contents under acidic conditions (pH 5.5). The scale bar is 100 nm.

The -Mn-O- bonds are known to be stable under neutral and basic pH, but quite sensitive in the reductive and mildly acidic environment, which are the characteristics of TME compared to normal tissues 33, 38. In the TME, the -Mn-O- bonds within the framework of silica shells are expected to be disrupted, which then further accelerates the biodegradation of Mn-ZGOCS nanocapsules. As extensively reported, the pH of the tumor extracellular microenvironment is about 7.2-6.5 while that of intracellular early endosome and lysosome can reach 6.2-5.0 30. Moreover, the tumor tissue and tumor cytosol commonly feature a reductive nature with GSH concentrations at least 4-folds higher than that in normal tissues 29, 51. Therefore, phosphate-buffered saline (PBS) with different pH values (7.4 and 5.5) and GSH concentrations (5.0 mM and 10.0 mM) is used to mimic the acidic and reducing condition of the TME, respectively. TEM and inductively coupled plasma optical emission spectrometer (ICP-OES) are used to monitor the degradation process of Mn-ZGOCS nanocapsules in the simulative TME (Figure 3). The nanostructure of Mn-ZGOCS nanocapsules shows no significant change in pH 7.4 PBS solution after 8 h (Figure 3A), indicating that Mn-ZGOCS nanocapsules are stable in the neutral environment 52. However, significant change can be found on the Mn-ZGOCS nanocapsules after 8 h incubation within an acidic PBS solution. Mn-ZGOCS nanocapsules present time-dependent degradation behavior in acidic solutions due to the breakup of Mn-O bonds and extraction of manganese from the framework of Mn-ZGOCS nanocapsules, which can be further demonstrated by ICP tests. The release of Mn ions from the framework of Mn-ZGOCS nanocapsules tends to be stable under the neutral environment but accelerated under the mild acidic condition (Figure 3B). Similar to the pH effect, the hollow structures of Mn-ZGOCS nanocapsules are quickly destroyed within 8 h incubation with PBS solution containing GSH (Figure 3C), and the biodegradation is GSH concentration-dependent. Corresponding ICP test results further demonstrate the ultrasensitive GSH-responsive biodegradation behavior of Mn-ZGOCS nanocapsules (Figure 3D). Moreover, the biodegradation behavior of Mn-ZGOCS nanocapsules under combined acidic (pH 5.5) and reducing conditions (GSH = 5.0 and 10.0 mM) are also recorded. It can be found that the biodegradation behavior of Mn-ZGOCS nanocapsules under such a combinatorial condition is much faster than the biodegradation in either a single acidic or reducing environment (Figure 3E). Such a quick structural collapse of Mn-ZGOCS nanocapsules can be strongly supported by the corresponding ICP test results (Figure 3F). More importantly, hollow structural collapses of Mn-ZGOCS nanocapsules can accelerate the dissolution of silica frameworks, and further release super-small ZGOCS NIR persistent luminescence nanoparticles, which can be validated to be cubic phase ZnGa_2_O_4_ by the corresponding SAED pattern in Figure S9. The results substantiate the biodegradation nature of Mn-ZGOCS nanocapsules, which is highly favored to the efficient drug release and clearance of these nanocapsules.

### TME-enhanced dual-mode imaging of Mn-ZGOCS-PEG nanocapsules

**Figure 4:**
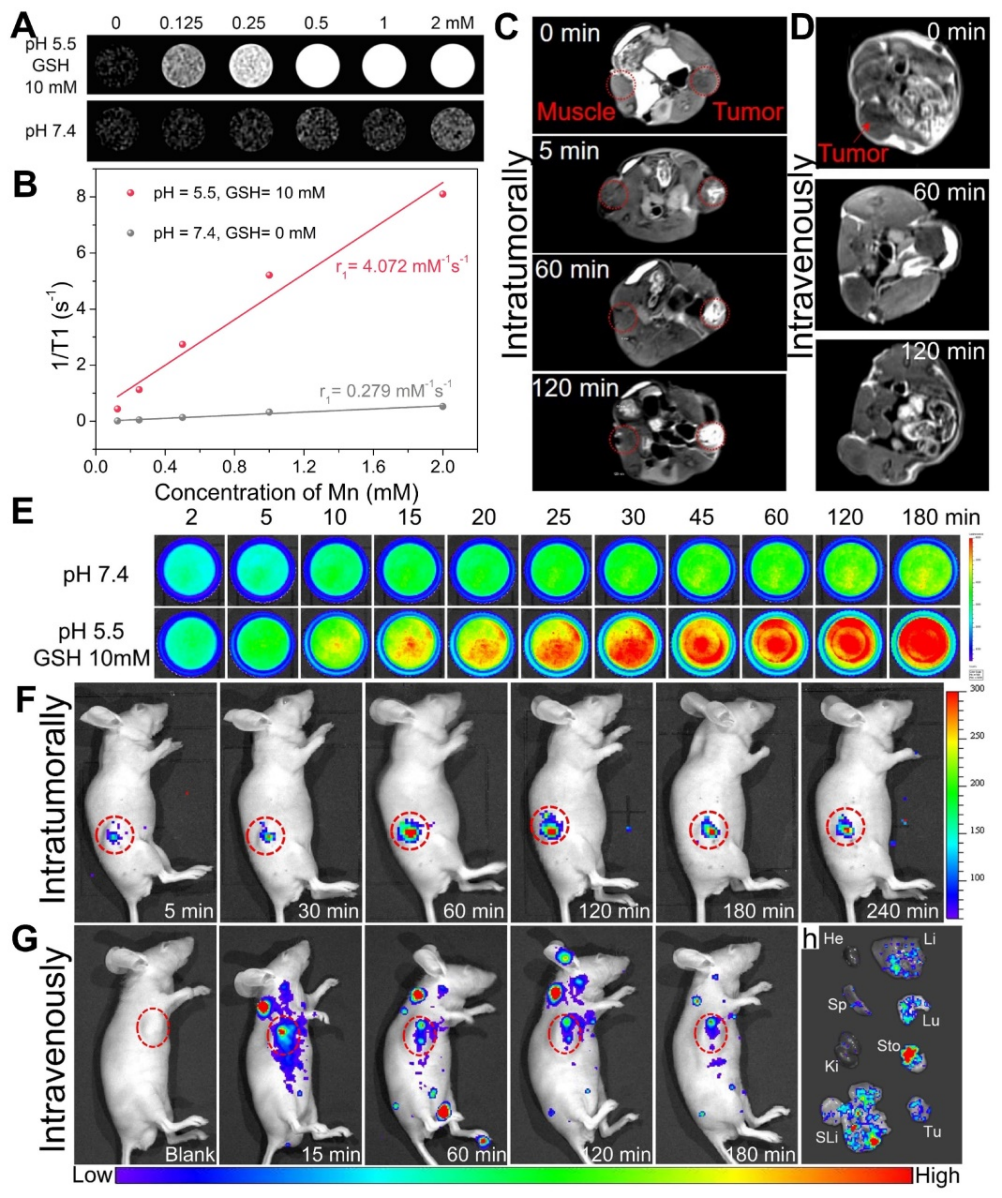
(A) T_1_-weighted in vitro MR images of Mn-ZGOCS-PEG nanocapsules with various concentrations after 8-hour incubation with neutral PBS (pH 7.4) or combined acidic and reductive PBS (pH 5.5, and GSH 10 mM), and (B) Corresponding T_1_-relaxation rate versus Mn ions concentration of Mn-ZGOCS nanocapsules. (C) Comparative T_1_-weighted MR images of LNCaP tumor-bearing nude mouse before and after subcutaneous injection of Mn-ZGOCS-PEG nanocapsules (0.5 mg/mL, 100 µL) within the normal muscle (left) and tumor tissues (right). (D) *In vivo* T_1_-weighted MR images of the mouse before and after intravenous injection of Mn-ZGOCS-PEG nanocapsules (0.5 mg/mL, 200 µL). (E) *In vitro* persistent luminescence images of Mn-ZGOCS-PEG nanocapsules (2 mg/mL, 2 mL) after various periods of incubation in PBS with various pH (7.4, 5.0) and GSH concentrations (0 and 10 mM). (F) *In vivo* persistent luminescence images of the mouse after intratumoral injection with Mn-ZGOCS-PEG nanocapsules (10 mg/mL, 20 µL) at different time points. (G) *In vivo* persistent luminescence images of the mouse after intravenous injection with Mn-ZGOCS-PEG nanocapsules (2 mg/mL, 200 µL) at different time points. (H) *Ex vivo* persistent luminescence images of various organs and tumor tissue at the 3 h after intravenous injection. He, Li, Sp, Lu, Ki, Sto, SLi, and Tu stood for heart, liver, spleen, lung, kidney, stomach, small and large intestine, and tumor, respectively.

Owing to five unpaired 3d electrons, Mn^2+^ is known to be a longitudinal (T_1_) relaxation agent for magnetic resonance (MR) imaging 53-55. As demonstrated, manganese within silica frameworks of Mn-ZGOCS-PEG nanocapsules is easily extracted in the combined acidic and reductive conditions, but stable under a neutral environment. Because of MR contrast effect is strongly related to the interaction between contrast agent and water protons, as-prepared Mn-ZGOCS-PEG nanocapsules with TME-responsive biodegradation behavior are expected to be tumor-specific MR contrast agent. Therefore, MRI properties of Mn-ZGOCS-PEG nanocapsules after being incubated for 8 h in neutral PBS (pH 7.4) and combined acidic and reductive PBS (pH 5.5, and GSH 10 mM) are characterized, respectively. The MR signals from Mn-ZGOCS-PEG nanocapsules are significant concentration-dependent positively enhanced in T_1_-weighted MR images in acidic and reductive conditions, whereas the signals of nanocapsules in the neutral condition appear to be much weaker (Figure 4A). Through the slope of the dependence of relaxation rate 1/T_1_ on Mn concentration (Figure 4B), the longitudinal relaxivity (r_1_) of Mn-ZGOCS-PEG nanocapsules after incubation in acidic and reductive PBS for 8 h is determined to be 4.072 mM^-1^s^-1^, which are much higher than that in the neutral condition (r_1_ = 0.279). Such an ultrasensitive TME-responsive MRI performance is due to the extraction of manganese from silica frameworks of Mn-ZGOCS-PEG nanocapsules, which enhance the interaction probability of paramagnetic Mn^2+^ with water protons. To evaluate the capabilities of such TME-responsive contrast agent for tumor-specific MR imaging, the same quantity of Mn-ZGOCS-PEG nanocapsules PBS (100 μL, 0.5 mg·mL^-1^) is injected into the tumor and the muscle on the opposite side for T_1_-weighted MR imaging, respectively (Figure 4C). It can be easily found that the enhancement of MR signals in the tumor area is much more than that in the muscle area. The increasing MR signal ratio of tumor area to muscle area provides direct evidence that as-prepared Mn-ZGOCS-PEG nanocapsules possess the capability of tumor-specific MR imaging (Figure S10). Furthermore, the *in vivo* T_1_-weighted MR images of tumor-bearing mice before and after intravenous injection of Mn-ZGOCS-PEG nanocapsules PBS (200 μL, 0.5 mg·mL^-1^) are performed. The *in vivo* T_1_-weighted MR images (Figure 4D) and the corresponding quantified MR signals in the tumor site (Figure S11) provide a clear representation of the time-dependent positive enhancement behavior of Mn-ZGOCS-PEG nanocapsules in MR imaging. The performance of tumor-enhanced MR imaging is ascribed to the synergetic contribution of passive tumor accumulation of nanocapsules via EPR effect and TME-triggered release of manganese, leading to high contrast with normal tissues.

On the other hand, the disorganization of Mn-ZGOCS-PEG nanocapsules in the acidic and reducing TME is expected to induce persistent luminescence recovery, permitting PL signal enhanced tumor imaging. Time-dependent emission spectra and *in vitro* PL imaging are tested to verify the luminescence recovery process of Mn-ZGOCS-PEG nanocapsules in the simulative TME. The luminescence intensity (Figure S12), the PL signal (Figure 4E), and the corresponding quantified signal to noise ratio (Figure S13) of Mn-ZGOCS-PEG nanocapsules are all significantly time-dependent enhanced in the acidic and reductive conditions (pH 5.5, and GSH 10 mM), whereas only a slight variation can be found in the neutral condition (pH 7.4). Faded color (Figure S14) and reduced absorbance in the NIR region (Figure S15) explain the characteristic luminescence recovery of Mn-ZGOCS-PEG nanocapsules in the simulative TME. Given the outstanding *in vitro* results, we then move forward to studying the performance of Mn-ZGOCS-PEG nanocapsules *in vivo*. As shown in Figure 4F, the NIR-PL signal in the tumor site is weak at 5 min after intratumoral injection of Mn-ZGOCS-PEG nanocapsules (20 μL, 10 mg·mL^-1^). As time goes on, this specific signal in the tumor site is gradually enhanced and tends to a plateau at 60 min, owing to the TME-responsive biodegradation behavior of Mn-ZGOCS-PEG nanocapsules (Figure S16). Moreover, NIR-PL imaging is conducted on tumor-bearing mice after intravenous injection of Mn-ZGOCS-PEG nanocapsules (200 μL, 2 mg·mL^-1^). A certain NIR-PL signal can be sustainably observed in tumor regions within 180 min, suggesting the efficient tumor accumulation of those Mn-ZGOCS-PEG nanocapsules (Figure 4G). Meanwhile, *ex vivo* NIR-PL images of major organs and tumors are collected at 180 min post-injection (Figure 4H). Strong NIR-PL signal found in tumor tissue indicates the high tumor uptake of Mn-ZGOCS-PEG nanocapsules, which can be supported by corresponding semiquantitative biodistribution analysis (Figure S17). Besides, the concentrations of element Zn (from ZGOCS) and Mn (from Mn-doped silica shell) within the organs and excreta are tested to evaluate the *in vivo* biodistribution and excretion analysis after the intravenous injection of Mn-ZGOCS-PEG nanocapsules into tumor-bearing mice. Massive Zn and Mn are found in the tumor, indicating the tumor accumulation of Mn-ZGOCS-PEG nanocapsules (Figure S18). Besides, the accumulation amount of Zn and Mn in feces (Figure S19) and urines (Figure S20) are significantly increased with time, demonstrating that Mn-ZGOCS-PEG nanocapsules can be effectively excreted out of the mice's body because of the quick biodegradation of Mn-ZGOCS-PEG nanocapsules. The TME-responsive dual-MR/NIR-PL-modality imaging capacity and efficient biological clearance indicate that Mn-ZGOCS-PEG nanocapsules have promise to be a clearable theranostic agent for imaging-guided tumor therapy.

### DOX delivery, cancer therapy, and biosafety evaluation of Mn-ZGOCS-PEG nanocapsules

**Figure 5:**
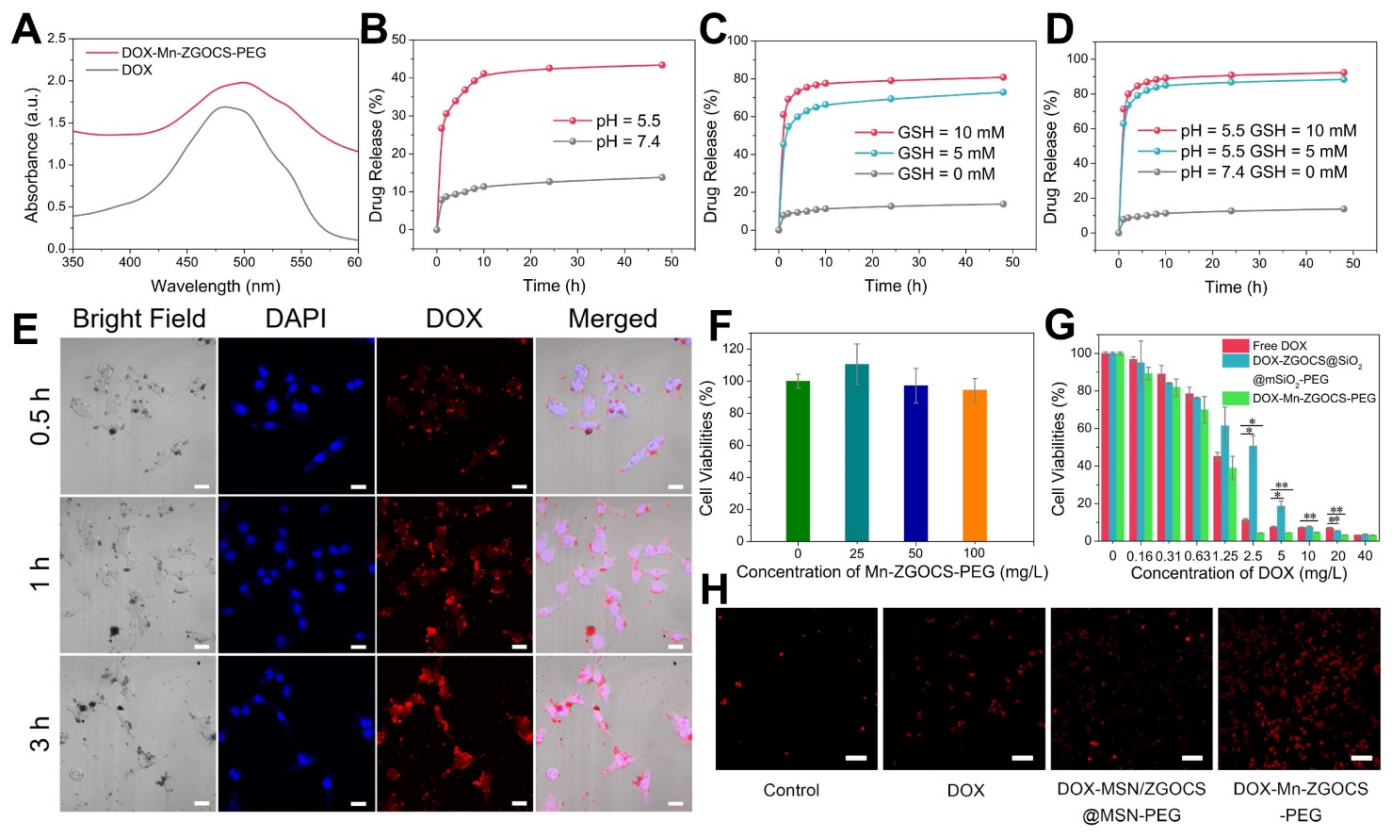
(A) UV-vis absorbance spectra of free DOX and DOX-Mn-ZGOCS-PEG nanocapsules. (B)-(D) Cumulative drug release profiles of the DOX-Mn-ZGOCS-PEG nanocapsules in PBS with various (B) pH values, (C) GSH contents, and (D) combined acidic and reductive conditions. (E) Confocal images of LNCaP cells incubated with DOX-Mn-ZGOCS-PEG nanocapsules at different time points. The blue and red represent DAPI and DOX fluorescence, respectively. The scale bar is 20 μm. (F) *In vitro* cell viabilities of LNCaP cells incubated with different concentrations of Mn-ZGOCS-PEG nanocapsules for 24 h. (G) Cytotoxicity of free DOX, DOX-ZGOCS@SiO_2_@mSiO_2_-PEG, and DOX-Mn-ZGOCS-PEG nanocapsules against LNCaP cells as a function of DOX concentrations for 24 h (*P < 0.05 and **P < 0.01). (H) Confocal images of LNCaP cells treated with PBS, DOX, DOX-ZGOCS@SiO_2_@mSiO_2_-PEG, and DOX-Mn-ZGOCS-PEG nanocapsules for 4 h, respectively, dyed with PI (red represent dead cells). The scale bar is 100 μm.

Inspired by the biodegradable behavior of Mn-ZGOCS-PEG nanocapsules, anticancer drug doxorubicin (DOX) is chosen as a model drug to study drug loading and release capacities of these nanocarriers. The UV-vis absorption spectrum verifies that the drug DOX is successfully loaded into the hollow structure of Mn-ZGOCS-PEG nanocapsules (Figure 5A). Through recording the absorbance of the DOX solution before and after the loading process (Figure S21), the DOX loading efficiency of Mn-ZGOCS-PEG nanocapsules is determined to be 47.3% in terms of the DOX standard curve (Figure S22). As a contrast, the DOX is also loaded into ZGOCS@SiO_2_@mSiO_2_-PEG nanocarriers with a loading efficiency of 26.4% (Figure S23), which is much lower than that of Mn-ZGOCS-PEG nanocapsules. Next, drug release behaviors of the DOX-loaded Mn-ZGOCS-PEG (DOX-Mn-ZGOCS-PEG) nanocapsules are studied. Compared with the low drug release rate of 13.8% at pH 7.4 after a period of 48 h, the cumulative release of DOX at pH 5.5 is speeded up to 43.4% after the same period, verifying a clear pH-dependent release behavior of the DOX-Mn-ZGOCS-PEG nanocapsules (Figure 5B). Notably, such a responsive release behavior is also confirmed in PBS with different GSH contents, where PBS containing GSH enables the quicker drug release (Figure 5C). The cumulative release of DOX in PBS containing GSH is increased to 72.9% and 80.8% at GSH concentrations of 5.0 and 10.0 mM, respectively. Moreover, the release speed of DOX is found to be much faster in concurrent acidic and reducing conditions (Figure 5D), owing to the fast shell biodegradation of Mn-ZGOCS-PEG nanocapsules. We next study the cellular uptake of DOX-Mn-ZGOCS-PEG nanocapsules and their intracellular DOX releasing behavior. Human prostate adenocarcinoma cells (LNCaP cells) are incubated with DOX-Mn-ZGOCS-PEG nanocapsules for 0.5, 1, and 3 h at 37 °C, and then imaged by a confocal laser scanning microscopy (CLSM). As shown in Figure 5e, distinct red fluorescence representing DOX, which is loaded in the Mn-ZGOCS-PEG nanocapsules, is found in cancer cells and enhanced with prolonging of incubation time, indicating that as prepared nanocarriers could be efficiently ingested by the cells. It is also noteworthy that obvious accumulation of DOX is found in the nucleus of cancer cells over time, confirming that DOX-Mn-ZGOCS-PEG nanocapsules can gradually release DOX intracellularly after cellular uptake. Then, the efficacy of DOX-Mn-ZGOCS-PEG nanocapsules as an efficient chemotherapy nano-agent is evaluated at the *in vitro* level. The *in vitro* cytotoxicity of the Mn-ZGOCS-PEG nanocapsules is firstly tested on LNCaP human prostate adenocarcinoma cells by CCK-8 assay (Figure 5F). No significant toxicity to LNCaP cells is observed after 24 h incubation with Mn-ZGOCS-PEG nanocapsules even at a concentration as high as 100 mg·L^-1^. Moreover, it is found that the intracellular GSH content is gradually decreased with the increase of Mn-ZGOCS-PEG nanocapsules concentration, indicating that the extraction of Mn ions is occurred in LNCaP cells and then triggers the intracellular biodegradation of Mn-ZGOCS-PEG nanocapsules (Figure S24). Upon loading DOX, the Mn-ZGOCS-PEG nanocapsules give good performance in cancer cell killing (Figure 5G). Compared to the free DOX, DOX-Mn-ZGOCS-PEG nanocapsules loaded equivalent concentration of DOX show higher lethality to cancer cells, indicating that Mn-ZGOCS-PEG nanocapsules regulated DOX delivery can enhance the anticancer effect of DOX on LNCaP cancer cells. It is worth noting that DOX-Mn-ZGOCS-PEG nanocapsules are found to be more effective in killing cancer cells than DOX-ZGOCS@SiO_2_@mSiO_2_-PEG. The enhanced therapeutic efficacy can be attributed to the quick release of DOX triggered by the quick intracellular biodegradation of Mn-ZGOCS-PEG nanocapsules. Furthermore, Propidium iodide (PI), which can dye dead cells with a red color, is used to assess the therapeutic efficacy of free DOX, DOX-ZGOCS@SiO_2_@mSiO_2_-PEG nanocarriers, and DOX-Mn-ZGOCS-PEG nanocapsules (Figure 5H) 12. Among them, DOX-Mn-ZGOCS-PEG nanocapsules cause the largest proportion of dead cells, demonstrating the best anticancer effectiveness of as prepared nanocarriers, which is following the results in Figure 5G.

**Figure 6:**
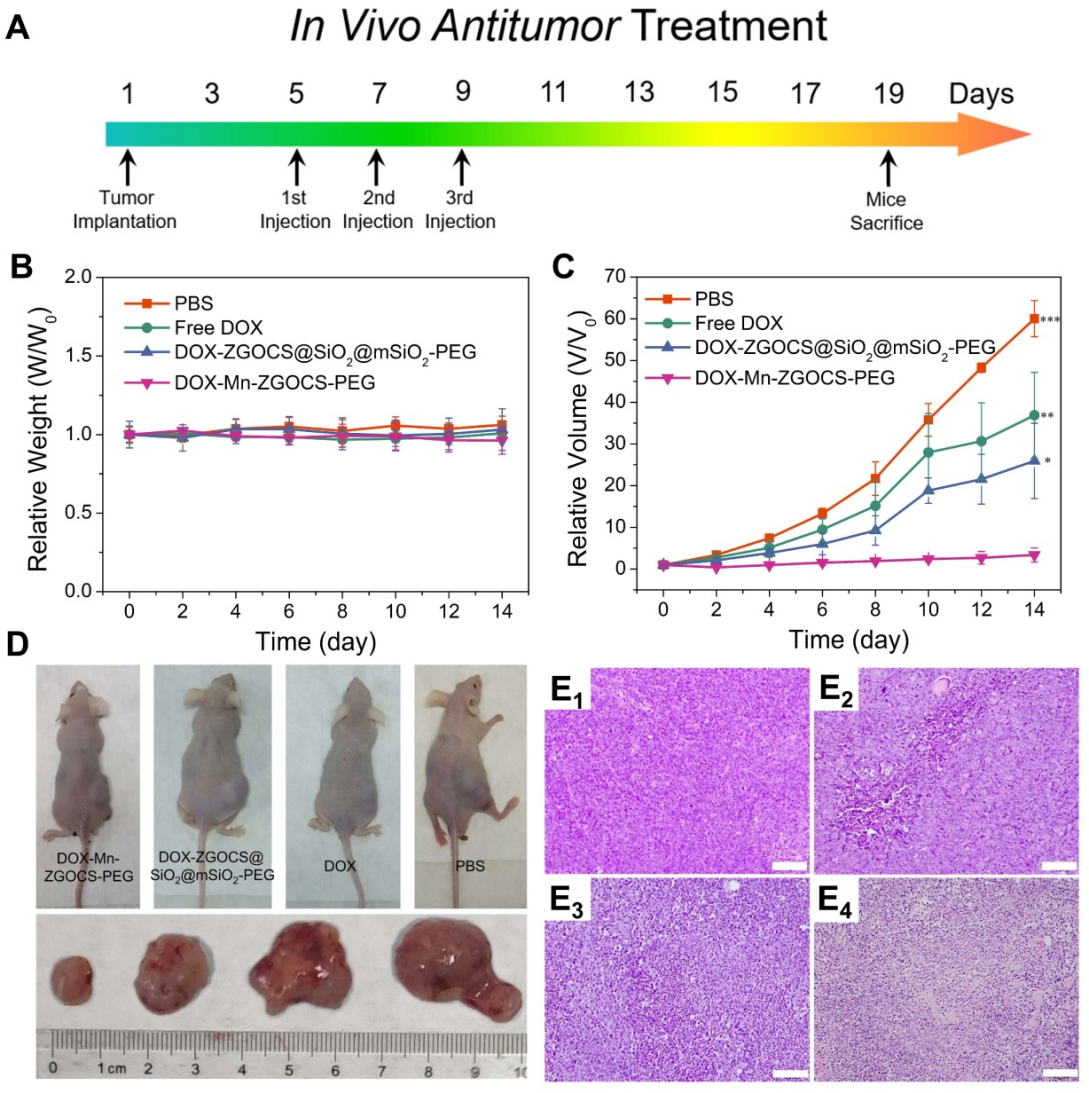
*In vivo* anticancer tumor effect of DOX-Mn-ZGOCS-PEG nanocapsules. (A) The therapeutic protocol of in vivo antitumor treatment. (B) Bodyweight changes in different groups of mice under various treatments. Bodyweight is recorded every other day and the relative weight is normalized to their initial weight. (n = 4) (C) Corresponding growth curves of different groups of mice under various treatments. The relative tumor volume is normalized to its initial size (n = 4, mean ± s.d., *P < 0.05, **P < 0.01, and ***P < 0.001). (D) Representative photographs of mice and excised tumors obtained on the 14th day after various treatments. (E_1 - 4_) H&E-stained images of LNCaP solid tumor tissues obtained on the 14th day after various treatments: 1, PBS (control); 2, Free DOX; 3, DOX-ZGOCS@SiO_2_@mSiO_2_-PEG; 4, DOX-Mn-ZGOCS-PEG nanocapsules at the equivalent DOX dose. The scale bar is 100 μm.

Encouraged by the above *in vitro* therapy results, the *in vivo* chemotherapy experiments are performed on LNCaP tumor xenograft nude mice. Before that, the hemolytic effects against red blood cells (RBCs) are evaluated on DOX, DOX-ZGOCS@SiO_2_@mSiO_2_-PEG nanoparticles, and DOX-Mn-ZGOCS-PEG nanocapsules, respectively. All three samples show negligible hemolytic effects, which implied high biosafety of these nanoparticles after intravenous injection (Figure S25). In the *in vivo* treatment experiments, LNCaP tumor-bearing mice are randomly divided into four groups: (1) PBS (control); (2) Free DOX; (3) DOX-ZGOCS@SiO_2_@mSiO_2_-PEG; and (4) DOX-Mn-ZGOCS-PEG. As the therapeutic proposal is shown in Figure 6A, nanomedicines are intravenously injected into mice at the equivalent DOX dose of 2.5 mg·kg^-1^. During the 14-days treatment, the mice in four groups have negligible weight fluctuations and no mice death is found, demonstrating few adverse effects of these treatments on the health of mice (Figure 6B and Figure S26). The tumor volume is also recorded to evaluate the antitumor efficacy (Figure 6C). Free DOX showed a negligible effect on tumor growth, likely owing to the insufficient tumor retention of DOX at such low doses. However, the intravenous administration of DOX-Mn-ZGOCS-PEG nanocapsules with the same DOX dose has significantly inhibited tumor growth. Moreover, the therapeutic efficacy of DOX-Mn-ZGOCS-PEG nanocapsules is stronger than DOX-ZGOCS@SiO_2_@mSiO_2_-PEG, which is under in vitro therapy results. Such a high tumor-inhibition effect is attributed to the excellent tumor retention of DOX-Mn-ZGOCS-PEG nanocapsules within tumor tissues and efficient release of DOX triggered by fast shell biodegradation in mild acidic/reducing TME. The photographs of mice and excised tumors obtained from a representative mouse in each group also confirm the highest antitumor efficacy of DOX-Mn-ZGOCS-PEG nanocapsules (Figure 6D). Furthermore, the severest tumor tissue necrosis can be found in the hematoxylin and eosin (H&E) staining images of tumor slices after the treatment with DOX-Mn-ZGOCS-PEG nanocapsules, maintaining good consistency with the above results (Figure 6E_1-4_). Besides, the histological analysis of the major organs of the mice, including the heart, liver, spleen, lung, and kidney, is also performed to evaluate the *in vivo* toxicity of DOX-Mn-ZGOCS-PEG nanocapsules (Figure S27). Following the systemic toxicity test standard (ISO 10993-11:2006), the acute and subacute systemic toxicity of DOX-Mn-ZGOCS-PEG nanocapsules is tested at 24 h and 14 d, respectively 56. Compared to the control mice, no obvious pathological abnormity is observed in the major organ tissues of the treated mice, confirming the good biocompatibility of DOX-Mn-ZGOCS-PEG nanocapsules for potential clinical trials.

## Conclusion

In summary, smart biodegradable theranostic NDDSs integrating ultra-small NIRPLNPs and hollow mesoporous Mn-doped silica shells (Mn-ZGOCS-PEG) are constructed for concurrent TME-responsive dual-MR/NIR-PL bioimaging and chemotherapy of tumors. The well-designed Mn-ZGOCS-PEG nanocapsules with a large hollow interior demonstrate advantages in highly efficient loading of the drug as well as precisely controlled drug release owing to the biodegradation of Mn-doped silica shell in either mildly acidic or reducing microenvironment of tumor tissues. Moreover, the TME induced manganese release from Mn-ZGOCS-PEG nanocapsules enables tumor-specific MR imaging, as well as enhanced autofluorescence-free PL imaging, resulted from the TME-responsive fluorescence recovery. More importantly, both *in vitro* and *in vivo* experiments confirm that our as-prepared Mn-ZGOCS-PEG nanocapsules possess excellent cancer cell killing ability and high tumor-inhibition efficacy without adverse effects or off-target toxicity. Together, these results show that our smart biodegradable Mn-ZGOCS-PEG nanocapsules hold tremendous translational potential for future clinical cancer therapy.

## Supplementary Material

Supplementary figures.
