# Supplementary material for: Biodegradable manganese engineered nanocapsules for tumor-sensitive near-infrared persistent luminescence/magnetic resonance imaging and simultaneous chemotherapy
Source: Theranostics. 2021 Jul 25;11(17):8448–63. doi: 10.7150/thno.59840 (PMC8344013; doi:10.7150/thno.59840)
Supplement: Supplementary file 1 — Supplementary figures. [file thnov11p8448s1.pdf]

Biodegradable manganese engineered nanocapsules for tumor-sensitive near-infrared persistent luminescence/magnetic resonance imaging and simultaneous chemotherapy

Rui Zou[1,2,3], Junwei Li[2], Ting Yang[1], Yong Zhang[1], Ju Jiao[1✉], Ka-Leung Wong[3✉], Jing Wang[2✉]

1. Department of Nuclear Medicine, The Third Affiliated Hospital of Sun Yat-sen University, 600 Tianhe Road, Guangzhou, Guangdong 510630, P.R. China
2. Ministry of Education Key Laboratory of Bioinorganic and Synthetic Chemistry, State Key Laboratory of Optoelectronic Materials and Technologies, KLGHEI of Environment and Energy Chemistry, School of Chemistry, Sun Yat-sen University, Sun Yat-Sen University, Guangzhou 510275, P.R. China
3. Department of Chemistry, Hong Kong Baptist University, Hong Kong S.A.R. 999077, P.R. China

✉ Corresponding authors: E-mail: jiaoju2@mail.sysu.edu.cn; ceswj@mail.sysu.edu.cn; klwong@hkbu.edu.hk

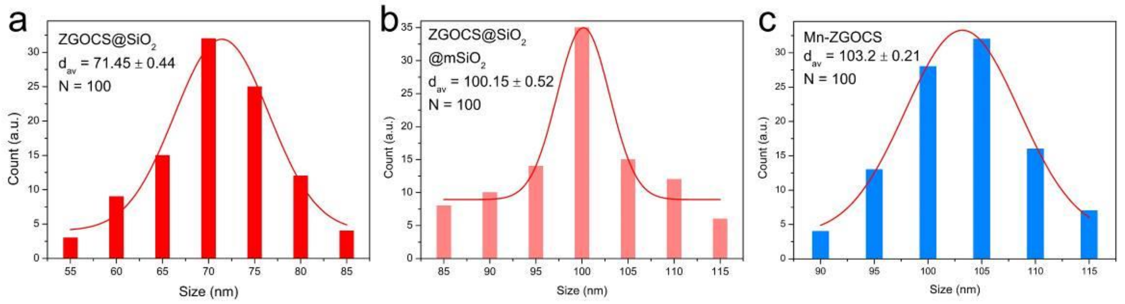

Figure S1. The size distribution of (a) $ZGOCS@SiO_2$ core nanoparticles, (b) $ZGOCS@SiO_2@mSiO_2$ nanoparticles, and (c) Mn-ZGOCS nanocapsules.

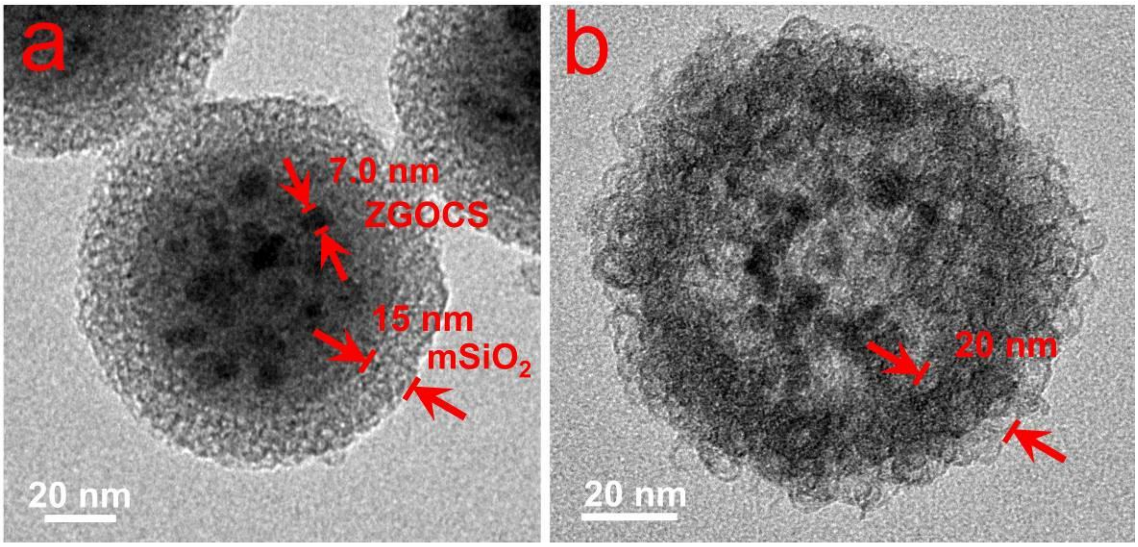

Figure S2. HRTEM images of (a) $ZGOCS@SiO_2@mSiO_2$ nanoparticles, (b) Mn-ZGOCS-PEG nanocapsules.

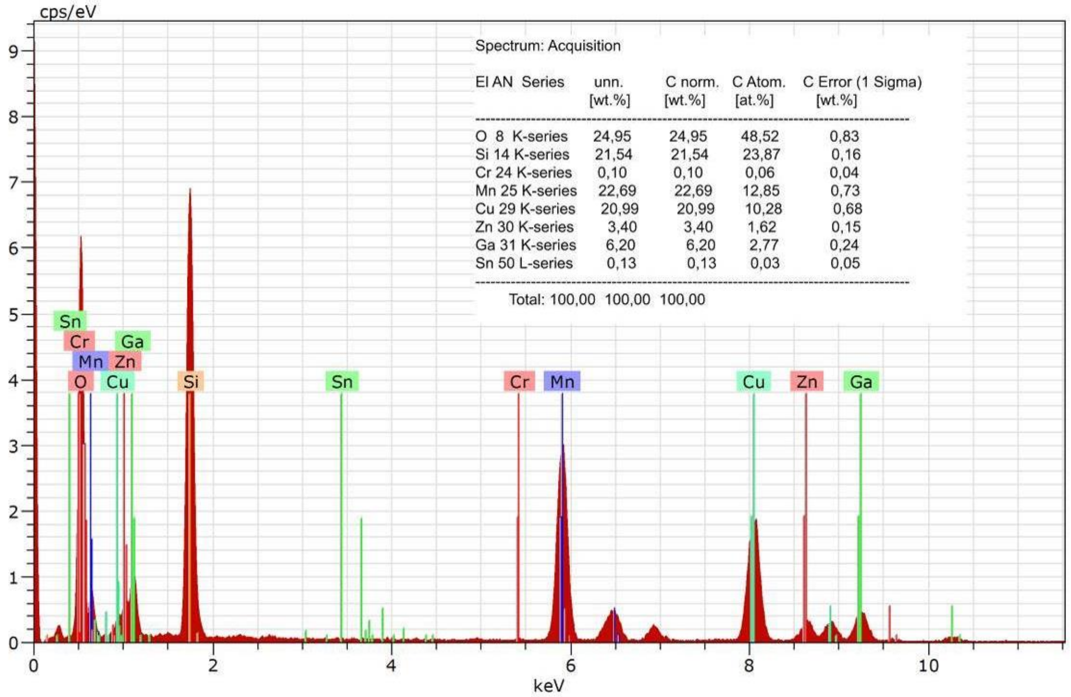

Figure S3. EDS spectrum of Mn-ZGOCS-PEG nanocapsules.

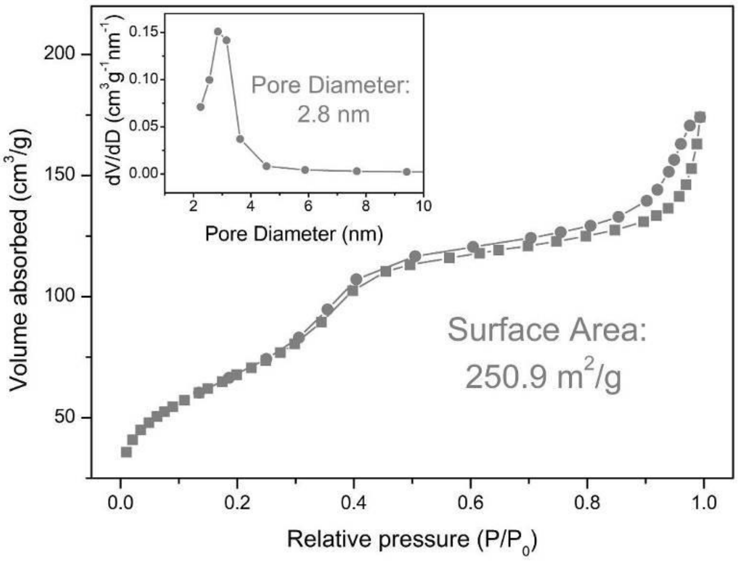

Figure S4. N_2 adsorption/desorption isotherm and pore size distributions (inset) of ZGOCS@SiO_2@mSiO_2 nanoparticles.

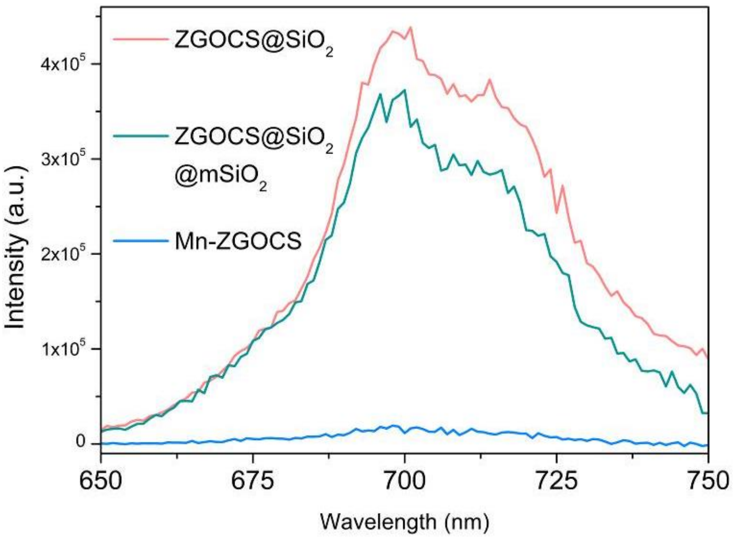

Figure S5. Afterglow emission spectra of ZGOCS@SiO_2, ZGOCS@SiO_2@mSiO_2 nanoparticles, and Mn-ZGOCS nanocapsules.

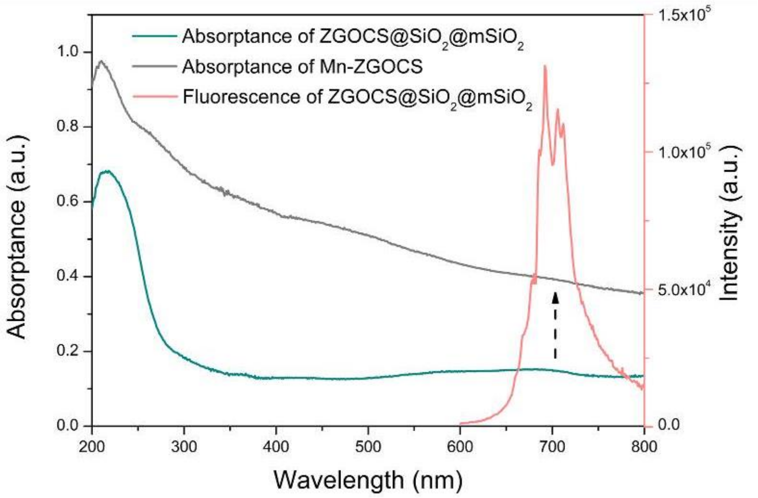

Figure S6. UV−vis absorbance spectra of ZGOCS@SiO_2@mSiO_2 and Mn-ZGOCS, and emission spectrum of the ZGOCS@SiO_2@mSiO_2. Due to the introduction of Mn into the silica framework of ZGOCS@SiO_2@mSiO_2 nanospheres, the absorption ranging from 600 nm to 800 nm is noticeably enhanced, resulting in luminescence quenching of Mn-ZGOCS nanocapsules.

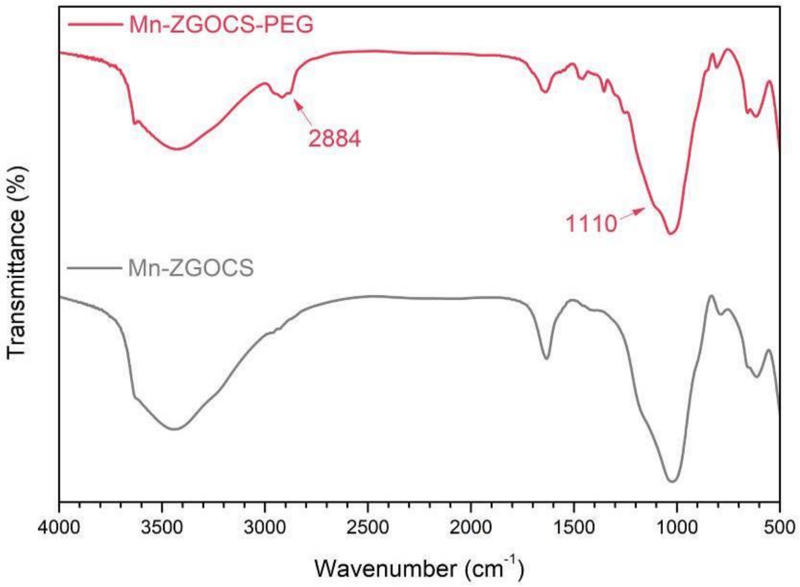

Figure S7. FT-IR spectra of Mn-ZGOCS (grey) and Mn-ZGOCS-PEG (red) nanocapsules.

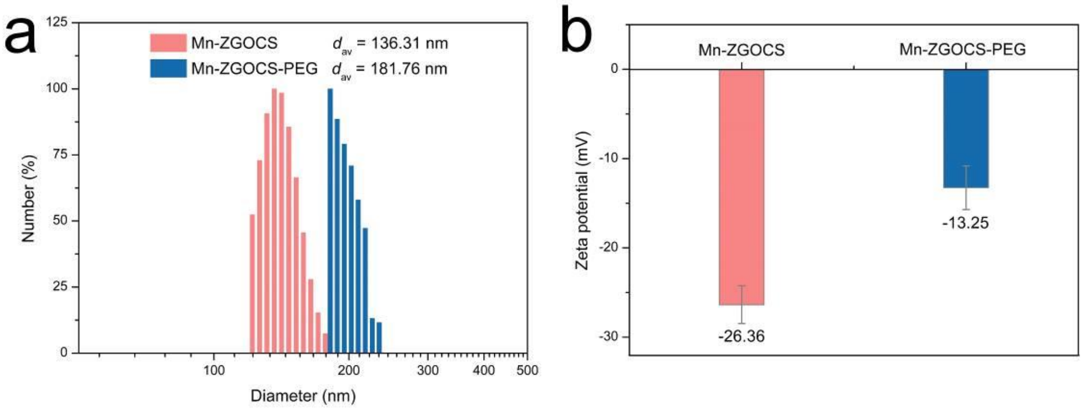

Figure S8. (a) The mean hydrodynamic diameter and (b) Zeta potential of Mn-ZGOCS and Mn-ZGOCS-PEG in saline.

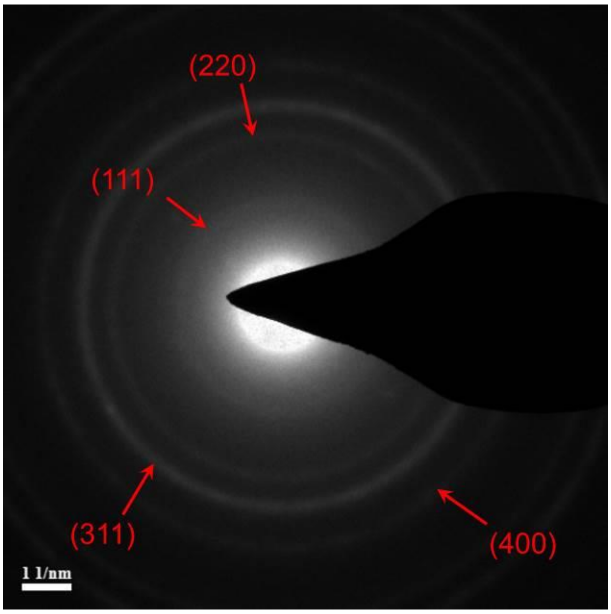

Figure S9. SAED pattern of Mn-ZGOCS nanocapsules after incubation with combined acidic and reductive PBS (pH 5.5, and GSH 10 mM) for 8 h.

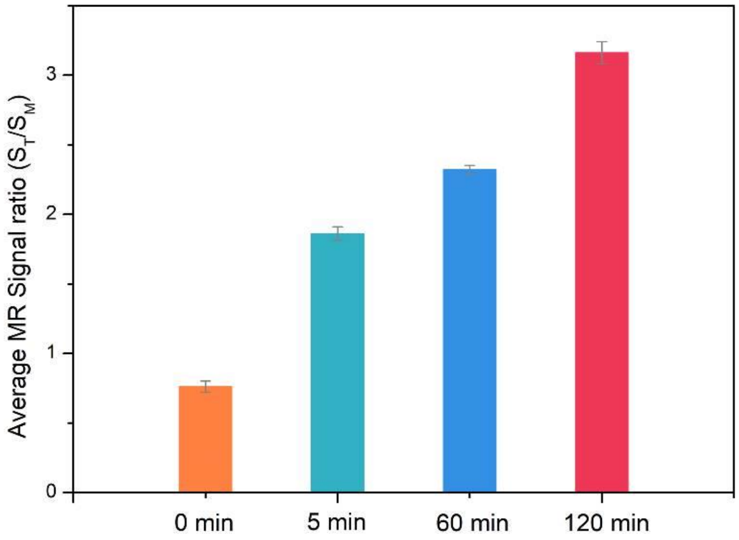

Figure S10. The ratio of quantified MR signals in tumor to in muscle before and after injection of Mn-ZGOCS-PEG nanocapsules.

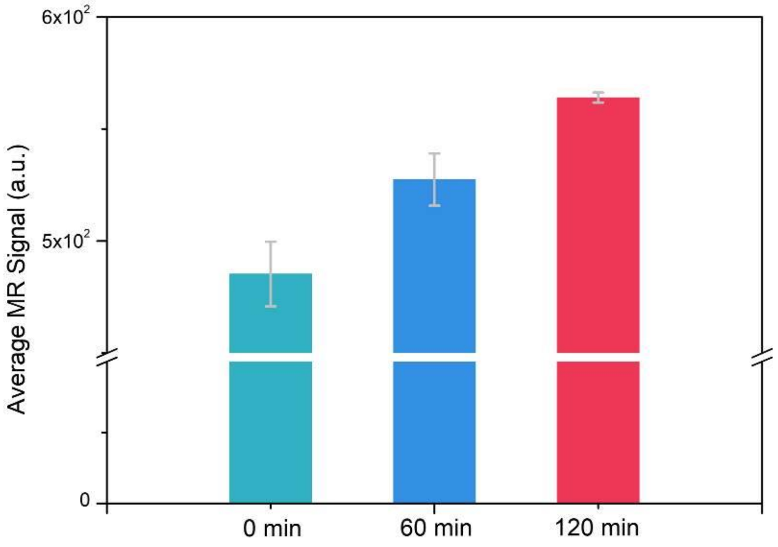

Figure S11. Comparison of quantified MR signals from in vivo T_1-weighted images in tumor site before and after the intravenous injection of Mn-ZGOCS-PEG nanocapsules.

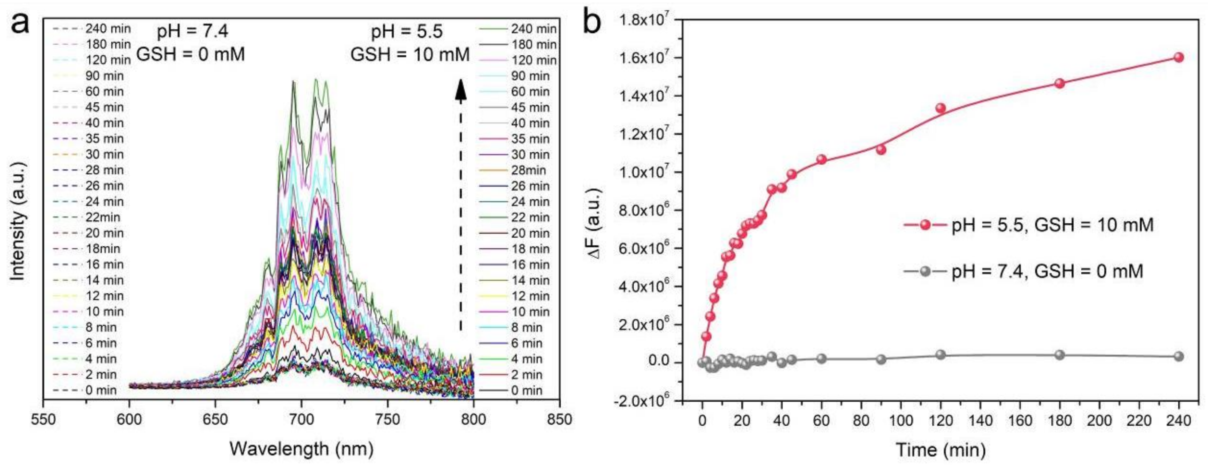

Figure S12. (a) Emission (excitation at 254 nm) spectra and (b) the corresponding recovered luminescent integral intensity of Mn-ZGOCS-PEG nanocapsules as a function of incubation time at various conditions. Dashed and solid present neutral PBS (pH 7.4) and combined acidic and reductive PBS (pH 5.5, and GSH 10 mM), respectively.

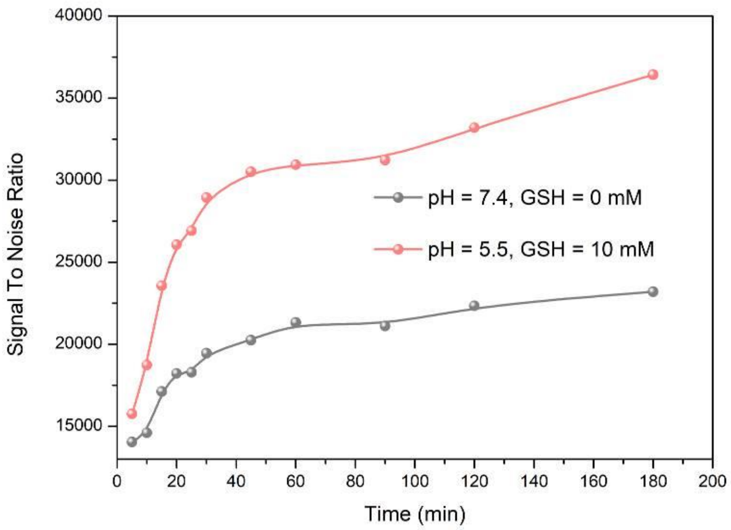

Figure S13. The signal-to-noise ratio of Mn-ZGOCS-PEG nanocapsules as a function of incubation time at various conditions.

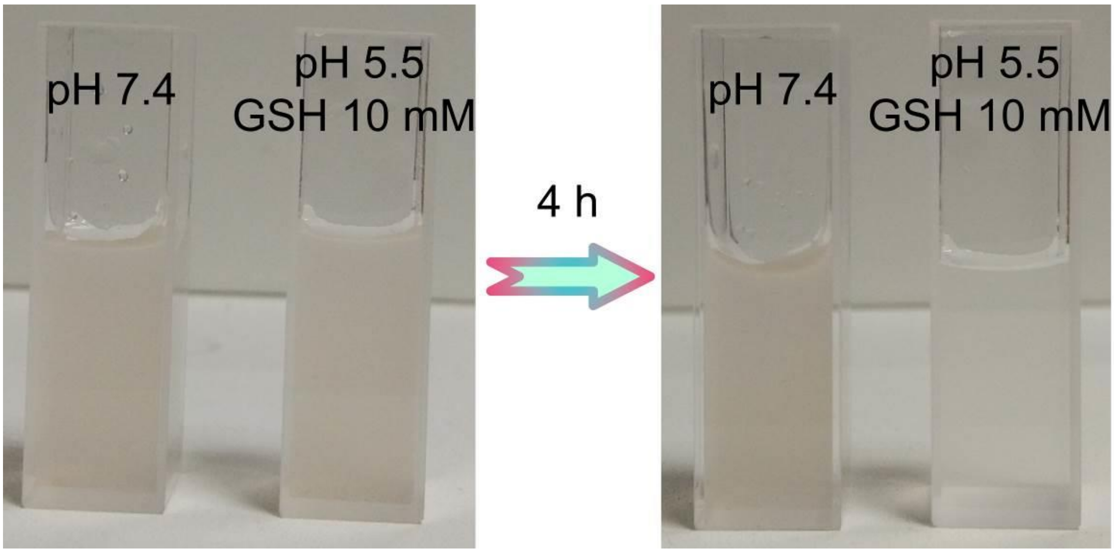

Figure S14. Digital photos of Mn-ZGOCS-PEG nanocapsules in neutral PBS (pH 7.4) and combined acidic and reductive PBS (pH 5.5, and GSH 10 mM) before and after 4 h of incubation. The color of Mn-ZGOCS-PEG nanocapsules solution is bleached after incubation with combined acidic and reductive PBS for 4 h.

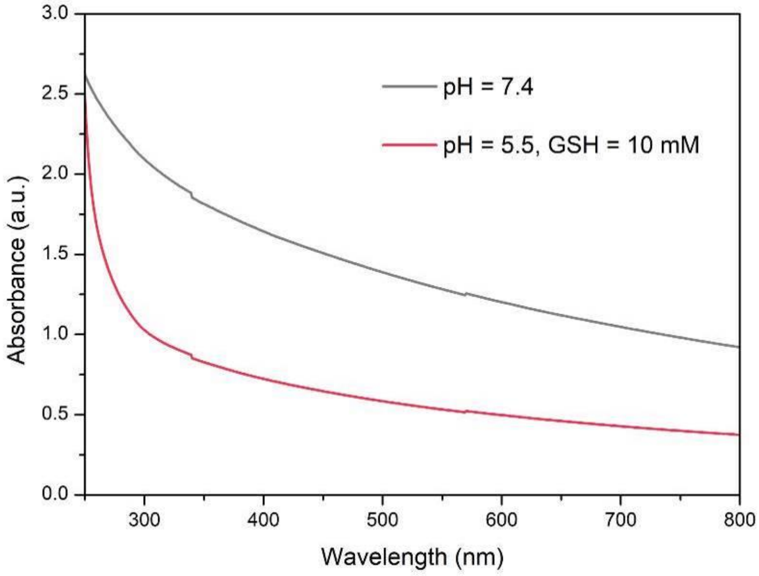

Figure S15. UV-vis absorbance spectra of Mn-ZGOCS-PEG nanocapsules in neutral PBS (pH 7.4) and combined acidic and reductive PBS (pH 5.5, and GSH 10 mM) before and after 4 h of incubation.

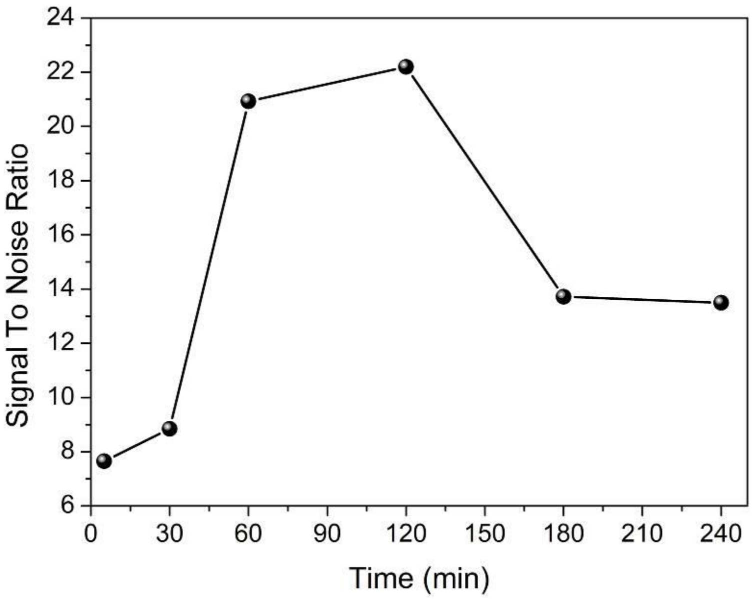

Figure S16. The ratio of quantified PL signals in tumor to in surrounding skin after intratumoral injection with Mn-ZGOCS-PEG nanocapsules (10 mg/mL, 20 μL) at different time points.

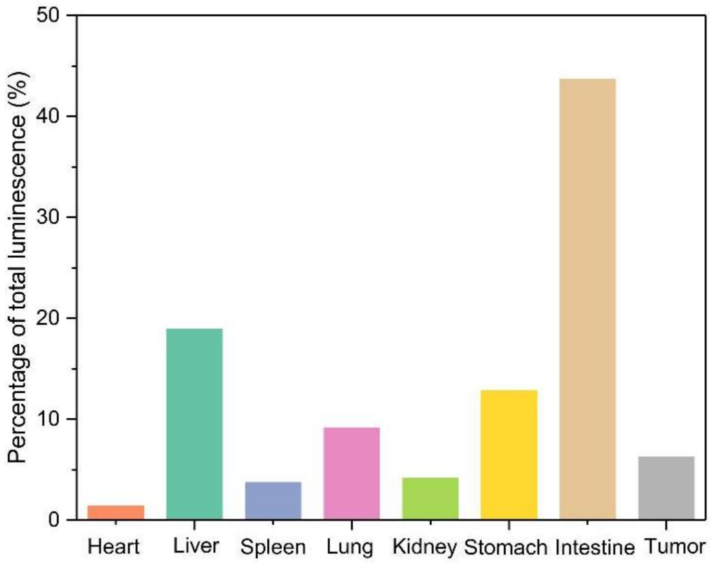

Figure S17. Semi-quantitative analysis of ex vivo persistent luminescence images in different organs and tumors.

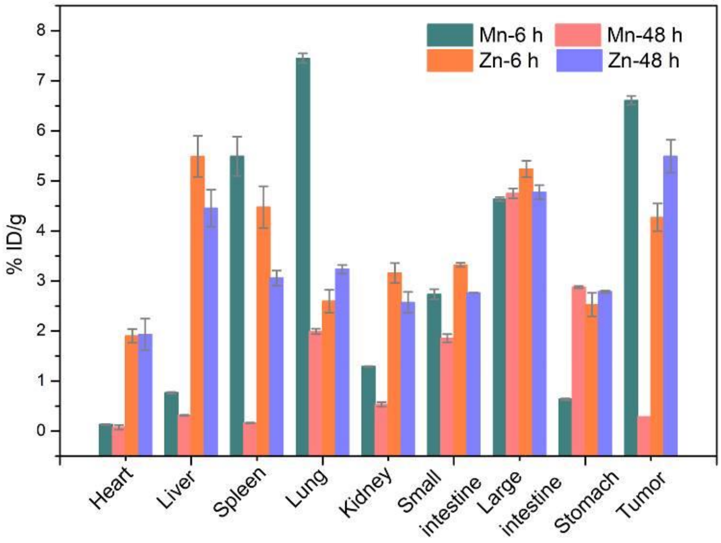

Figure S18. Biodistribution of element Mn and element Zn in the major organs of tumor-bearing mice at 6 and 48 h after intravenous injection of Mn-ZGOCS-PEG nanocapsules.

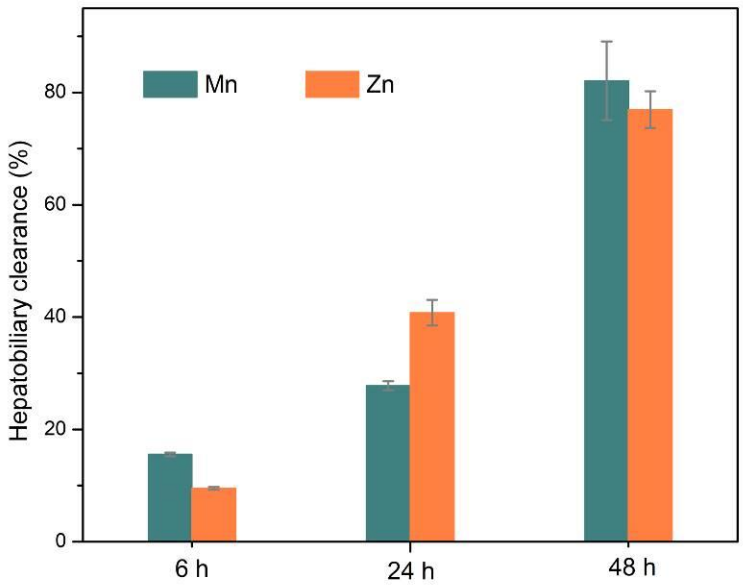

Figure S19. Accumulated element Mn and element Zn in the feces excreted out of tumor-bearing mice at 6, 24, and 48 h after intravenous injection of Mn-ZGOCS-PEG nanocapsules.

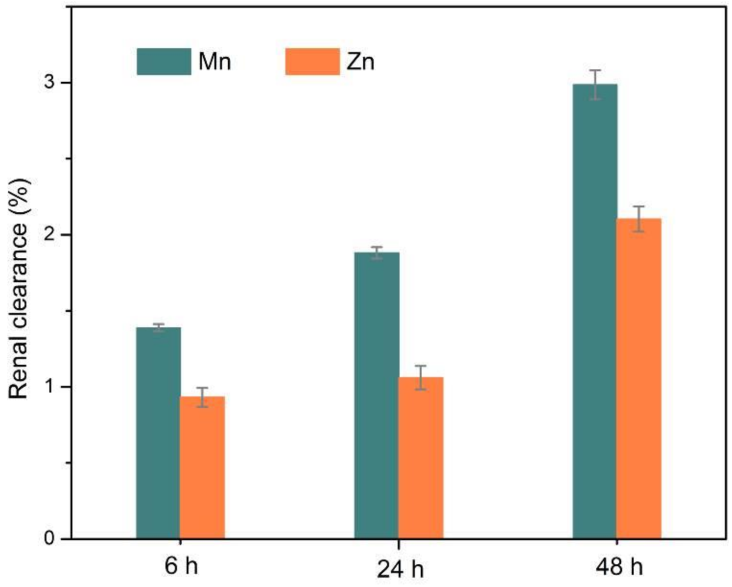

Figure S20. Accumulated element Mn and element Zn in the urine excreted out of tumor-bearing mice at 6, 24, and 48 h after intravenous injection of Mn-ZGOCS-PEG nanocapsules.

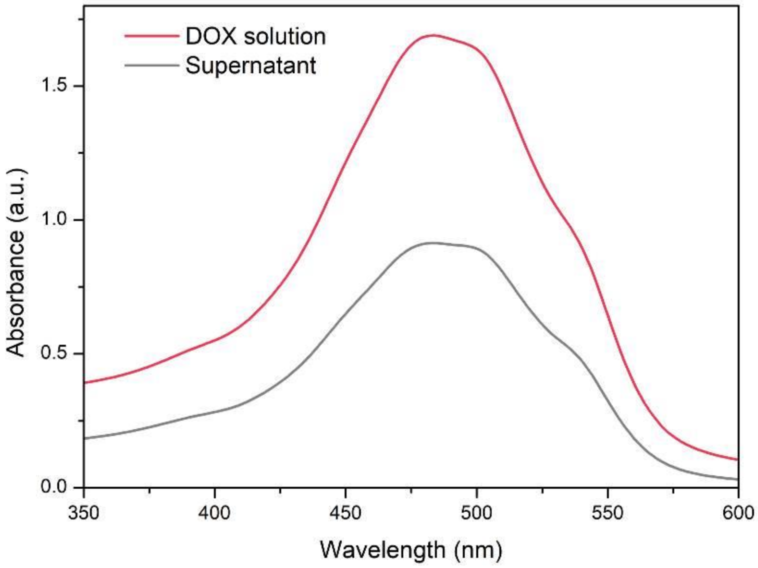

Figure S21. UV-vis absorbance spectra of the initial DOX solution and the supernatant obtained after the drug loading process with Mn-ZGOCS-PEG nanocapsules.

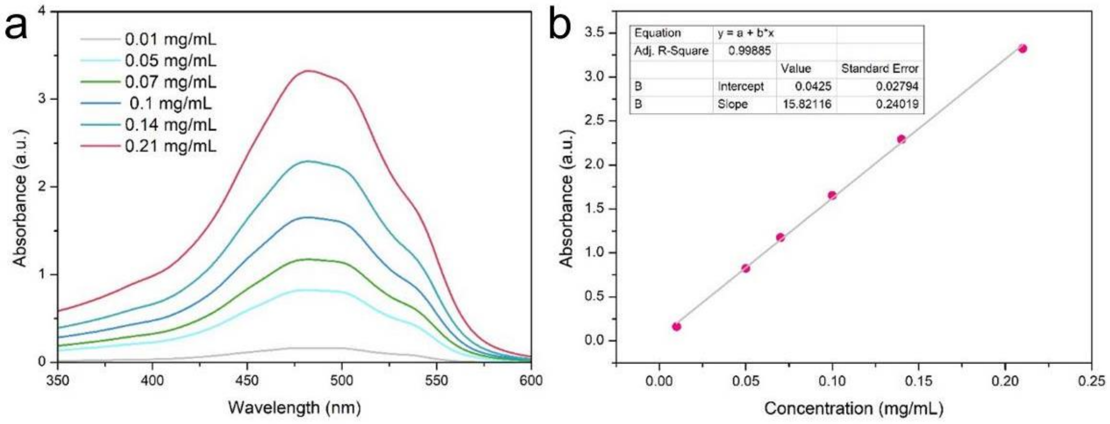

Figure S22. (a) UV-vis absorbance spectra of DOX solutions with various concentrations. (b) The standard curve of concentration-dependent DOX solutions absorbance at 480 nm.

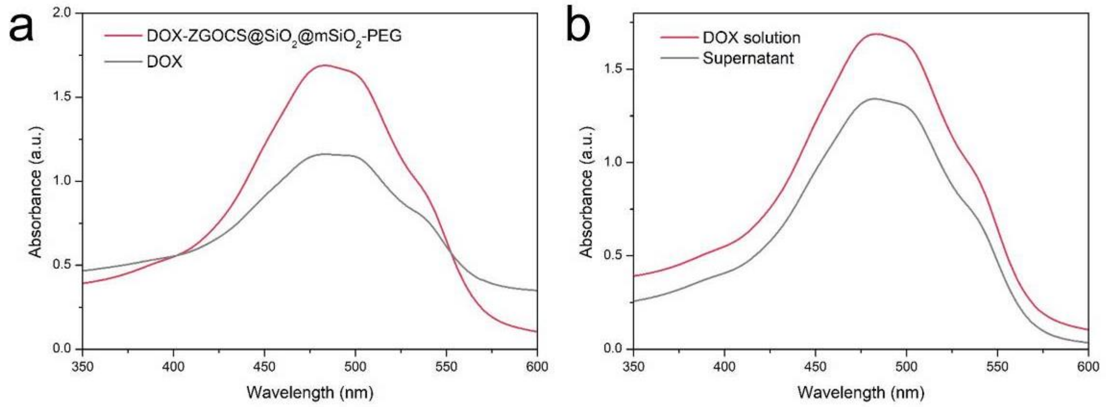

Figure S23. (a) UV-vis absorbance spectra of free DOX and DOX-ZGOCS@SiO_2@mSiO_2-PEG nanoparticles. (b) UV-vis absorbance spectra of the initial DOX solution and the supernatant obtained after the drug loading process with ZGOCS@SiO_2@mSiO_2-PEG nanoparticles.

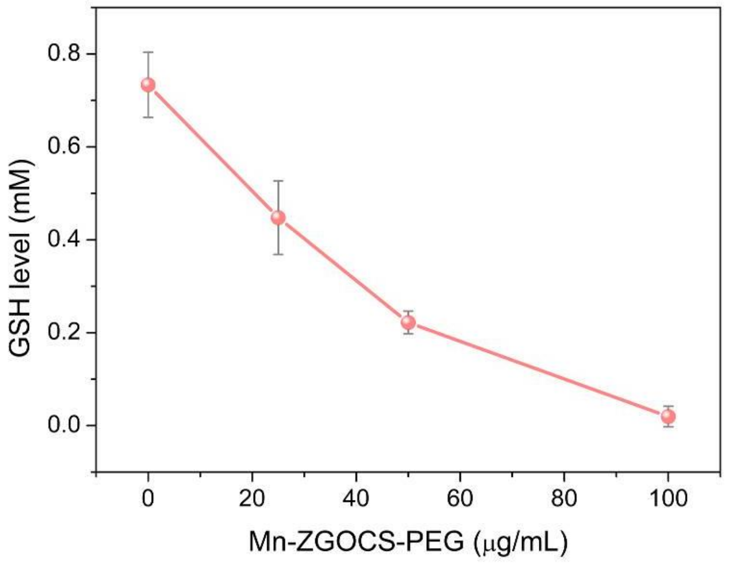

Figure S24. Intracellular GSH levels of LNCaP cells after the treatment with various concentrations of Mn-ZGOCS-PEG nanocapsules.

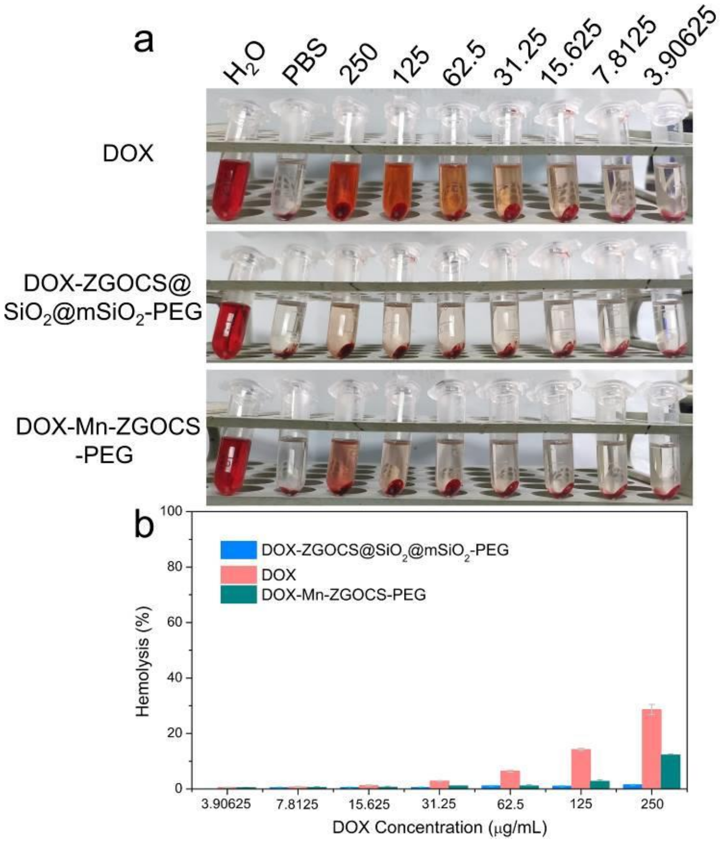

Figure S25. Blood hemolysis analysis of DOX, DOX-ZGOCS@SiO_2@mSiO_2-PEG nanoparticles, and DOX-Mn-ZGOCS-PEG nanocapsules. (a) photographs and (b) The corresponding hemolytic percentages of RBCs after various treatments. PBS and deionized water are set as the negative and positive control, respectively. It was found that the RBCs are unbroken and corresponding hemolytic percentages are low, indicating that all three samples exhibit excellent biosafety to RBCs. Due to the intrinsic absorption of DOX, the hemolytic percentages of DOX is abnormally high.

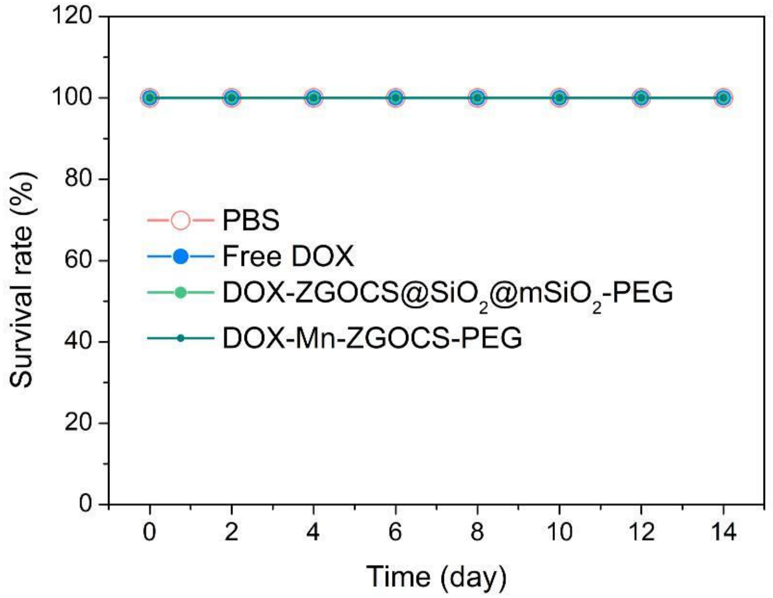

Figure S26. Survival curves in different groups of mice under various treatments.

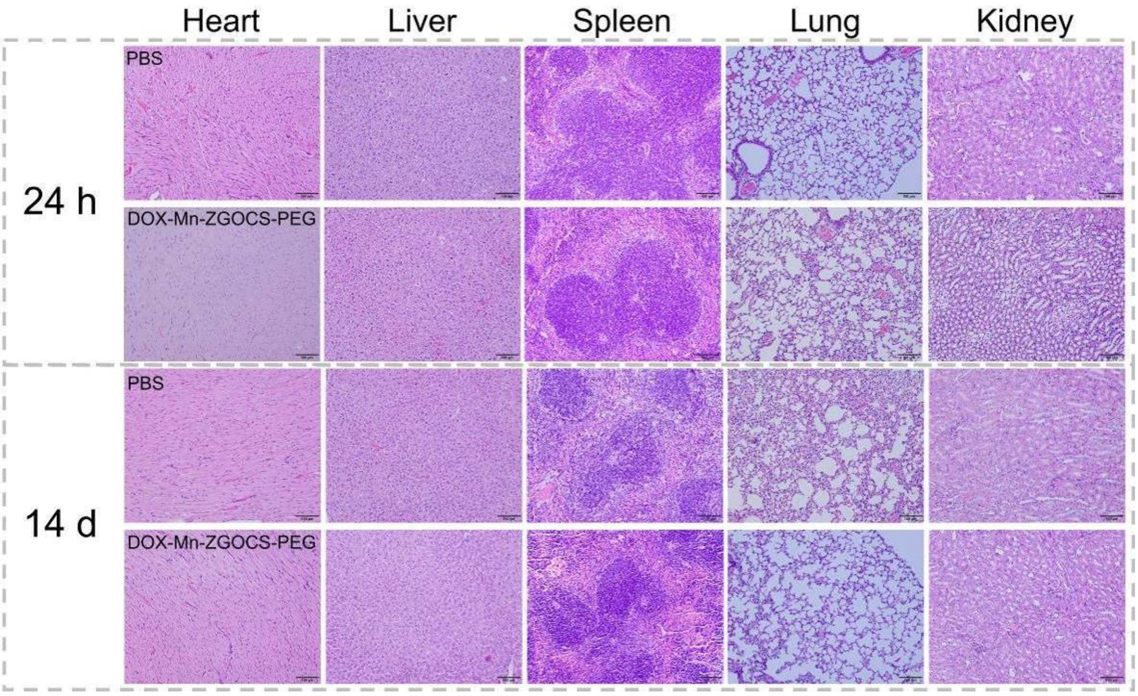

Figure S27. H&E-stained images of major organs (heart, liver, spleen, lung, kidney) from mice after the treatment with PBS (control) and DOX-Mn-ZGOCS-PEG nanocapsules, respectively. (Scale bars: 100 μm.) The treated mice are dissected after 24 hours and 14 days of treatment, respectively. No noticeable abnormality is observed in these major organs.
